# Macrophage Interaction with *Paracoccidioides brasiliensis* Yeast Cells Modulates Fungal Metabolism and Generates a Response to Oxidative Stress

**DOI:** 10.1371/journal.pone.0137619

**Published:** 2015-09-11

**Authors:** Juliana Alves Parente-Rocha, Ana Flávia Alves Parente, Lilian Cristiane Baeza, Sheyla Maria Rondon Caixeta Bonfim, Orville Hernandez, Juan G. McEwen, Alexandre Melo Bailão, Carlos Pelleschi Taborda, Clayton Luiz Borges, Célia Maria de Almeida Soares

**Affiliations:** 1 Laboratório de Biologia Molecular, Instituto de Ciências Biológicas, Universidade Federal de Goiás, Goiânia, Goiás, Brazil; 2 Unidad de Biología Celular y Molecular, Corporación para Investigaciones Biológicas (CIB), Medellín, Colombia; 3 Grupo de Investigación MICROBA, Escuela de Microbiología, Universidad de Antioquia, Medellín, Colombia; 4 Facultad de Medicina, Universidad de Antioquia, Medellín, Colombia; 5 Instituto de Ciências Biomédicas, Departamento de Microbiologia, Laboratório de Micologia, Universidade de São Paulo, São Paulo, Brazil; 6 Departamento de Ciências Fisiológicas, Universidade Federal do Amazonas, Manaus, Amazonas, Brazil; Newcastle University, UNITED KINGDOM

## Abstract

Macrophages are key players during *Paracoccidioides brasiliensis* infection. However, the relative contribution of the fungal response to counteracting macrophage activity remains poorly understood. In this work, we evaluated the *P*. *brasiliensis* proteomic response to macrophage internalization. A total of 308 differentially expressed proteins were detected in *P*. *brasiliensis* during infection. The positively regulated proteins included those involved in alternative carbon metabolism, such as enzymes involved in gluconeogenesis, beta-oxidation of fatty acids and amino acids catabolism. The down-regulated proteins during *P*. *brasiliensis* internalization in macrophages included those related to glycolysis and protein synthesis. Proteins involved in the oxidative stress response in *P*. *brasiliensis* yeast cells were also up-regulated during macrophage infection, including superoxide dismutases (SOD), thioredoxins (THX) and cytochrome c peroxidase (CCP). Antisense knockdown mutants evaluated the importance of CCP during macrophage infection. The results suggested that CCP is involved in a complex system of protection against oxidative stress and that gene silencing of this component of the antioxidant system diminished the survival of *P*. *brasiliensis* in macrophages and in a murine model of infection.

## Introduction

Paracoccidioidomycosis (PCM) is a human systemic mycosis that is restricted to Latin America, particularly Brazil, Colombia and Venezuela [1]. The disease is caused by members of the *Paracoccidioides* genus. These fungi are thermo-dimorphic species that grow as mycelium under saprobic conditions at 22–26°C, or as pathogenic yeast at 36°C [2]. The saprobic form lives in the soil and reaches the lung alveoli upon the inhalation of spores or mycelia fragments by the host, where they interact with epithelial cells and alveolar macrophages [3]. The fungus converts to the yeast pathogenic form at body temperature [4].

Macrophages constitute one of the primary defense mechanisms against infection by *P*. *brasiliensis;* thus, PCM is considered to be a classic granulomatous disease [5,6]. As a facultative intracellular pathogen, *P*. *brasiliensis* can persist inside macrophages. Microscopic studies showed that *P*. *brasiliensis* multiplied intracellularly in macrophages and this could be a factor in pathogenicity [5,6,7,8].

A characteristic of macrophages is the production of copious amounts of oxidants during the respiratory burst process [9]. The oxidative burst, a reaction characterized by increased oxygen uptake and ROS (reactive oxygen species) production, challenges parasite viability [10]. The most important ROS and RNS (reactive nitrogen species) generated inside the phagolysosome are nitric oxide (NO•), peroxynitrite (ONOO-), superoxide anion radical (O2-•), and hydroxyl radical (•OH) [11]. In particular, the success of pathogens is based on their resistance to nitrosative and oxidative stresses, and other environmental attacks [12]. During the infection process, *Paracoccidioides* spp. can cope with the RNS and ROS generated during the respiratory burst of phagocytic cells, as suggested by the arsenal of related transcripts [13]. Indeed, members of the *Paracoccidioides* spp. complex express a powerful antioxidant defense system in the presence of ROS-mediated oxidative stress [14]. Proteomic analysis demonstrated that the fungus presented a global activation of antioxidant enzymes, such as catalases, superoxide dismutases, cytochrome c peroxidase and thioredoxin when exposed to H_2_O_2_. The activation of the pentose phosphate pathway, a great source of cellular reducing power in the form of NADPH, suggested that there was a shift in the metabolism of yeast cells [14]. Response to nitrosative stress was also evaluated in *Paracoccidioides* sp. [15]. In this sense, a RNA approach to silence the gene encoding cytochrome c peroxidase depicted mutants highly sensitive to nitrosative stress [15]. Enzymes acting in the oxidative stress response also played a role in the nitrosative stress. Additionally, we demonstrated that carbon starvation exerted a strong effect on *Paracoccidioides* sp.. This stress, which is presumably similar to that found in the macrophage environment, evoked a shift to a starvation mode as determined at the transcriptional and proteomic levels. The metabolic alterations included an increase in gluconeogenesis and fermentative ethanol production, activation of fatty acids and amino acid degradation; these strategies are likely used by the pathogen to persist under this type of stress [16].

As described above, studies have begun to elucidate the complex transcriptional and translational programs that *Paracoccidioides* spp. use to survive when exposed to host-like conditions [14,15,16]. Tavares and co-workers (2007) showed that *P*. *brasiliensis* regulated the expression of 119 classified genes during phagocytosis; these genes were primarily associated with glucose and amino acid limitation, cell wall construction and oxidative stress [17].

One method for analyzing the *P*. *brasiliensis*-macrophage encounter should be the identification of alterations in the proteome, as the fungus is undergoing phagocytosis. In this way, in the present study, we assessed the response of *P*. *brasiliensis* to macrophage phagocytosis by employing high throughput proteomic analysis. The importance of the regulated proteins for the survival of *P*. *brasiliensis* within macrophages was inferred. This study demonstrated that the knockdown of cytochrome c peroxidase resulted in decreased survival of *P*. *brasiliensis* inside macrophages and affected fungal survival in the liver and spleen of infected mice.

## Materials and Methods

### Fungal strains and growth conditions

*P*. *brasiliensis* isolates *Pb*18 [18] and Pb339 [19] in the yeast form were used in this work. The yeast cells were cultivated in BHI medium, containing 4% glucose (w/v) for 48 h at 36°C under agitation.

### Interaction assay of *P*. *brasiliensis* and J774 1.6 macrophage cells

J774 1.6 macrophages (Rio de Janeiro Cell Bank—BCRJ/ UFRJ, accession number 0273) were used for the phagocytosis assays. The J774 1.6 cells were cultured in RPMI medium containing bovine fetal serum 10% (v/v) and MEM non-essential amino acid solution (Sigma Aldrich, Missouri, USA) at 36°C and 5% CO_2_ until completely confluence. The phagocytosis assay was performed in 12-well polypropylene plates (Greinner Bio-One, USA). A total of 10^6^ J774 1.6 macrophages were plated per well in RPMI medium containing IFN-γ (1U/mL) (Sigma Aldrich) and incubated for 24 h at 36°C and 5% CO_2_ for adherence and activation. Then, the medium was replaced to a fresh RPMI medium containing IFN-γ (1U/mL) and 5x10^6^ Pb18 yeast cells per well were added to the macrophages, resulting in a yeast:macrophage cell ratio of 5:2, since the doubling time for J7741.6 cells is around 20-24h. The cells were incubated for 24 h at 36°C and 5% CO_2_. Then, macrophages were lysed with water and fungal cells recovered. The control condition was obtained by incubating 5x10^6^ yeast cells per well in RPMI medium containing IFN-γ (1U/mL) for 24 h at 36°C and 5% CO_2_.

### Evaluation of phagolysosome maturation

The maturation of phagolysosomes was assessed using the Lysotracker probe red DND99 (Life Technologies Carlsbad, USA) according to the manufacturer’s instructions. Briefly, the macrophage cells were labeled with 75 nM of the Lysotracker probe for 60 min, prior to fungal infection, to avoid labeling of *P*. *brasiliensis* cells. The interaction assay of J774 1.6 macrophages with *P*. *brasiliensis* was performed as described, in a 6-well polypropylene plate, with cover slip. After, the cells were washed three times with sterile phosphate buffered saline (PBS), and incubated with 2 mg/mL of fluorescent brightener (Calcofluor white M2R, Sigma Aldrich) for 1 h, to label the fungal cells. Macrophage J774 1.6 cells were used as the control. The cover slips were fixed with 4% paraformaldehyde (Sigma Aldrich) for 1 h, washed three times with sterile PBS removed and photographed at bright field, at 579/599 nm for Lysotracker and at 395/420 nm for Calcofluor White, using an Axioscope A1 fluorescence microscope (Carl Zeiss).

### Preparation of protein extracts

The interaction assay of *P*. *brasiliensis* yeast cells with macrophages was performed as described above. Then, the cells were washed three times with PBS and the macrophages were lysed by the addition of sterile water. The lysates were centrifuged at 8,000 x g for 10 min. The obtained pellet containing *P*. *brasiliensis* yeast cells was washed three times with water. The pellet was ressuspended in a solution containing 20 mM Tris-HCl, pH 8.8, and 2 mM CaCl_2_ [20], and the protein extraction was performed in BeadBeater equipment (BioSpec, Bartlesville, USA) in tubes containing 200–500 μm of acid-washed glass beads (Sigma Aldrich) in equal volume of fungal pellet. Control cells were obtained by incubating *P*. *brasiliensis* yeast cells in RPMI medium. The obtained protein extracts were quantified using the Bradford reagent (Sigma Aldrich) with bovine serum albumin (BSA) (Sigma Aldrich) as the standard.

### Digestion of protein extracts and nano-ESI-UPLC-MS^E^ analyses

A total of 100 μg of each protein extract was used for trypsin digestion as previously described [21,22]. Briefly, 10 μL of 50 mM ammonium bicarbonate buffer, pH 8.5, was added to the samples, which were treated with 0.2% RapiGEST SF Surfactant (v/v) (Waters, Milford, MA, USA) and incubated in a dry bath at 80°C for 15 min. The samples were reduced with 100 mM DTT (GE Healthcare, Piscataway, NJ, USA) at 60°C for 30 min, and alkylated with 300 mM iodacetamide (GE Healthcare, Piscataway, NJ, USA) at room temperature for 30 min. Then, 20 μL of trypsin (50 ng/mL) (Promega, Madison, WI, USA) was added to digest the samples at 37°C in a dry bath for 16 h. To cleave and precipitate the RapiGEST reagent, 20 μL of trifluoroacetic acid (TFA) solution 5% (v/v) was added to the samples, followed by incubation for 90 min at 37°C. The supernatants were dried in a speed vacuum (Eppendorf, Hamburg, Germany) for 5 h. All obtained peptides were suspended in 100 μL of a solution containing 20 mM of ammonium formiate and 200 fmol/μL of PHB (Rabbit Phosphorylase B) (Waters Corporation, Manchester, UK) (MassPREP protein). Nanoscale LC separation of tryptic peptides was performed using a nanoACQUITY system (Waters) equipped with two reverse phase columns working in basic and acidic conditions. The first column was a nanoEase BEH130 C18 (1.7 μm, 100 μm x 100 mm; Waters, USA), and the second was a NanoAcquity UPLC column BEH 130 C18 (1.7 μm, 100 μm × 100 mm; Waters, USA). Mass spectrometry analysis was performed on a Synapt G1 MS (Waters, USA) equipped with a nanoelectronspray source and two mass analyzers: a quadrupole and a time-of-flight (TOF) operating in TOF V-mode. Data were obtained using the instrument in the MS^E^ mode, which switches the low energy (6 V) and elevated energy (20–40 V) acquisition modes every 0.4 s. Samples were analyzed from three replicates.

### Data processing and protein identification

The MS data obtained using the label-free MS^E^ protocol was processed using the ProteinLynx Global Server (PLGS) version 2.4 (Waters). The data were subjected to automatic background subtraction, deisotoping and charge state deconvolution. After processing, each ion comprised an exact mass-retention time (EMRT) that contained the retention time, intensity-weighted average charge, inferred molecular weight based on charge and *m/z*, and the deconvoluted intensity. Then, the processed spectra were searched against *Paracoccidioides brasiliensis* Pb18 protein sequences (Broad Institute; http://www.broadinstitute.org/annotation/genome/paracoccidioides_brasiliensis/MultiHome.html) together with random sequences. The mass error tolerance for peptide identification was under 50 ppm. The identified proteins showed a minimum of 1 matched peptide and 5 fragments, with at least 2 fragments belonging to the same peptide. Moreover, modifications such as methionine oxidation and serine, threonine and tyrosine phosphorylation were considered. A protein that showed a variance coefficient of 0.08 and that was detected in all replicates was used to normalize the protein expression levels in the samples (accession number: PADG_11272). The comparison of protein abundance was performed based on the average intensity value of the top three ionized tryptic peptides of this internal standard protein and was used to convert the average intensity of the analyte peptides to the corresponding absolute quantity of proteins loaded onto the column. Expression^E^ informatics v.2.5.2 was used for proper quantitative comparisons. The identified proteins were organized by the expression algorithm, into a statistically significant list, corresponding to induced and reduced regulation ratios between the infected and control groups. The mathematical model used to calculate the ratios was a part of the Expression algorithm inside the PLGS software from the Waters Corporation [23]. The minimum repeat rate for each protein in all replicates was 2.

Quality parameter such as the dynamic range of the experiments, peptide detection type, and mass accuracy were calculated for each condition using the software MassPivot and SpotFire as previously described [21]. Proteins that presented 50% differences in expression values compared to the control were considered to be regulated.

### Construction of the cytochrome c peroxidase (CCP) knockdown mutant

The antisense-RNA (aRNA) strategy was used as previously described [24]. Briefly, DNA from *Pb*339 yeast cells was obtained as described [15,25] and used to obtain the aRNA of *Pb*339 cytochrome c peroxidase (*ccp*). The oligonucleotides as*ccp*-sense 5’CCGCTCGAGCGGGATAAGGAAACTGGAACTGGAG 3’ and as*ccp*-antisense, 5’GGCGCGCCCCTGAGAGTGACCACGCTG 3’ were synthesized to amplify aRNA from DNA (PADG_03163; Accession number obtained in the *Paracoccidioides* database available at http://www.broadinstitute.org/annotation/genome/paracoccidioides_brasiliensis/MultiHome.html). Plasmid construction, *Agrobacterium tumefaciens* transformation and transformants selection was performed as described [15]. *P*. *brasiliensis* yeast cells were also transformed with the empty parental vector pUR5750 (EV) as a control for the assays performed in this study. Investigation of *Pbccp* gene expression was performed after consecutive sub culturing by quantitative real-time PCR. The interaction assay of two silenced mutants (*Pb*ccp-aRNA1 and *Pb*ccp-aRNA2) with J774 1.6 cells was performed using a yeast:macrophage ratio of 5:1. The control cells used in this assay were yeast cells from wild type *Pb*339 and *Pb*18 yeast cells transformed with the empty vector in the same yeast:macrophage ratio. The interaction assay using the silenced mutants *Pb*ccp-aRNA1 and *Pb*ccp-aRNA2 and the controls was performed in triplicate. Statistical analysis was performed using Student’s *t-*test. Results presenting *p-*values < 0.01 were considered statistically significant.

### Quantitative real-time PCR (qRT-PCR)

The evaluation of transcriptional levels was performed by qRT-PCR. Total RNA was extracted using Trizol (TRI Reagent, Sigma-Aldrich, St. Louis, MO, USA) and mechanical cell rupture using the Mini-Beadbeater (Biospec Products Inc., Bartlesville, OK, USA). Then, *in vitro* reverse transcription (SuperScript III First-Strand Synthesis SuperMix; Invitrogen, Life Technologies) was performed, and the cDNAs were subjected to qRT-PCR using the SYBR green PCR master mix (Life Technologies, Foster City, CA, USA) in the StepOnePlus real-time PCR system (Life Technologies). The expression values were calculated using the transcript encoding alpha tubulin (XM_002796593) as the endogenous control in the silencing mutation experiment and the transcript encoding beta tubulin (XM_002794440) in the *P*. *brasiliensis* interaction with macrophage cells experiment. The oligonucleotides used in the qRT-PCR to confirm the proteomic data were: beta tubulin sense: 5’-GATAACGAGGCTCTGTATGATA-3’; beta tubulin atsense: 5’ATGTTGACGGCGAGTTTGCG-3’; pbpase sense: 5’-GCCACTGGTGACTTTACCCT-3’; pbpase atsense: 5’-CATCTCCGGTGGTATTTGCG-3’; ccp RT sense: 5’-CTTTGACGACCGCGAGATTG-3’; and ccp RT atsense: 5’-GACCGTTCCACTTTCTCCAG-31.

The oligonucleotides used in the CCP silencing mutation experiments were: alpha tubulin sense: 5’-ATGAAACGGCAAATCCCACCA-3’; alpha tubulin antisense 5’- ACAGTGCTTGGGAACTATACC -3’; ccp-sense: 5’ CTTTGACGACCGCGAGATTG 3’ and ccp-antisense 5’ GACCGTTCCACTTTCTCCAG. The qRT-PCR reaction was performed in triplicate for each cDNA sample, and a melting curve analysis was performed to confirm the detection of a single PCR product. The relative standard curve was generated using a pool of cDNAs collected from all conditions serially diluted from 1:5 up to 1:125. The relative expression levels of the transcripts of interest were calculated using the standard curve method for relative quantification [26].

### Evaluation of *Pbccp*-aRNA sensitivity to oxidative stress

The *P*. *brasiliensis* cell strain *Pb*339 silenced with antisense technology for cytochrome c peroxidase (*Pbccp*-aRNA1 and *Pbccp*-aRNA2) was tested for sensitivity to oxidative stress. The cells were cultured as described above. A total of 10^5^ and 10^6^ cells/mL were plated onto solid Fava Netto’s medium containing 40 or 80 mM menadione. A total of 10^5^ and 10^6^ cells/mL from *Pb*18 and *Pb*339, respectively, were plated onto solid Fava-Netto’s medium with and without menadione as a control. *P*. *brasiliensis* strain *Pb*339 transformed with the binary empty vector (EV) was also used as control. The plates were incubated at 36°C for 7 days.

### BALB/c mouse infections

Mice were inoculated intraperitoneally with 10^7^ *P*. *brasiliensis* yeast cells from the WT, EV and *Pb*ccp-aRNA strains as previously described [27]. A total of 3 animals were used to each condition. After 7 days of infection, the animals were euthanized in CO_2_ chamber and mouse spleens and livers were removed and homogenized in 5 mL of sterile 0.9% (w/v) NaCl. The homogenized samples were plated in quintuplicate on brain heart infusion agar supplemented with 4% (v/v) fetal calf serum and 2% (w/v) glucose. The plates were incubated at 36°C, and colony forming units (CFUs) were determined after 10 days. The infection assay was performed in triplicate, and statistical analysis was performed using Student’s *t-*test. Results presenting *p-*values < 0.01 were considered statistically significant.

### Ethics Statement

The animal work conducted was reviewed and approved by Comissão de Ética no Uso de Animais of the Universidade Federal de Goiás-CEUA/UFG, license number 1922011. The animal care and use protocol is adhered to the Conselho Nacional de Controle de Experimentação Animal-CONCEA.

## Results

### Evaluation of phagolysosome maturation

**Fig 1:**
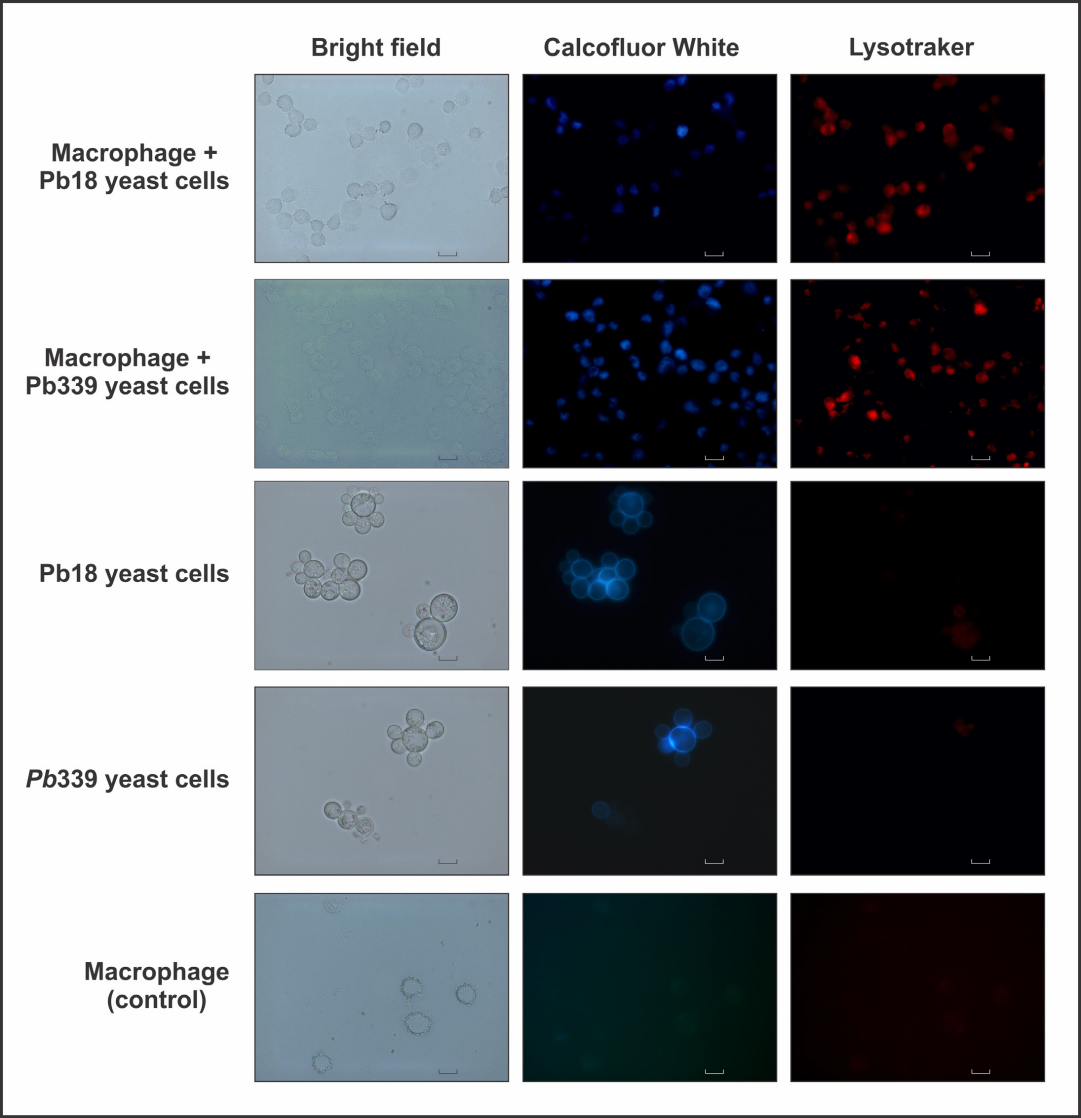
Interaction of *P*. *brasiliensis* yeast cells with macrophages and evaluation of phagolysosome maturation. The interaction assay was performed using two *P*. *brasiliensis* isolates (*Pb*18 and *Pb*339) and are shown in the lanes named macrophages + *Pb*18 yeast cells and macrophages + *Pb*339 yeast cells, respectively. The pictures were taken in bright field (shown in the bright field column), at 395/420 nm for Calcofluor probe (shown in the Calcofluor white column) and at 579/599 nm for Lysotracker probe (shown in the Lysotracker column). Fungal control cells are shown in the lanes named *Pb*18 yeast cells and *Pb*339 yeast cells. Control macrophage cells were also performed and are shown in the lane named macrophage (control). All representative pictures were taken using an Axioscope microscope (Carl Zeiss) and magnified 1000X.

Phagocytosis progression was evaluated by monitoring phagolysosome maturation with a lysosome marker as depicted in Fig 1. Phagolysosome maturation was observed using the Lysotracker probe to identify acidified phagolysosomes. The phagocytosis of *P*. *brasiliensis* yeast cells (*Pb*18 and *Pb*339) by J774 1.6 macrophages, induced phagolysosome maturation by acidification (Fig 1). Control was also performed with cells from these two *P*. *brasiliensis* isolates (*Pb*18 and *Pb*339). The fungal cells were visualized in Fig 1 using Calcofluor white, which does not stain J774 1.6 control cells. Control with J774 1.6 macrophages without fungal cells, was also performed as well as fungal cells from the two *P*. *brasiliensis* isolates without macrophages. The low pH provides an optimal environment for phagolysosomal enzymes and is an essential host strategy for the killing of most pathogens [28].

### Proteomic analysis

The resulting NanoUPLC-MS^E^ protein and peptide data generated by the PLGS analysis are shown in S3–S5 Files The experiments resulted in the identification of 7,845 peptides (4,461 and 3,384 in the control cells and those obtained after macrophage infection, respectively). A rate of 57.5% of the identified peptides were obtained from peptide match type data in the first pass and 6.5% from the second pass. A total of 17% of the peptides were identified by a missed trypsin cleavage, and an in-source fragmentation rate of 9% was observed (S3 File).

S4 File depicts the accuracy of the m/z fragment matches in the database. A total of 97.2% of the peptides were assigned with up to 15 ppm m/z of error. S5 File shows the abundant dynamic range of the identified proteins; the distribution of protein concentrations comprised 3 orders of magnitude. A total of 308 differentially expressed proteins were identified in *P*. *brasiliensis* yeast cells derived from infected macrophages and are depicted in S1 and S2 Files. A fold-change difference in the protein level of 50% in comparison with the control cells was used to identify the regulated proteins. A total of 139 proteins were positively regulated during macrophage infection (S1 File), while 179 proteins were down-regulated (S2 File).

Most of the up-regulated proteins (S1 File, S6 File, panel A) were related to amino acid metabolism (10.9% of the total), cell rescue, defense and virulence (10.9% of the total) and molecules involved in protein synthesis (7.8% of the total).

The most regulated functional classes in the down-regulated proteins included protein synthesis (17.9% of the total) and amino acid metabolism (11.7%). A total of 9.6% of the identified proteins were related to energy production, including enzymes acting in glycolysis, the tricarboxylic acid pathway and the electron transport chain, as well as glyoxylate and methylcitrate cycle components.

S6 File depicts the functional classes of the upregulated proteins (panel A) and down regulated proteins (panel B).

### *P*. *brasiliensis* adapts to the macrophage milieu by reprogramming its metabolism to produce glucose and inhibiting protein synthesis

**Fig 2:**
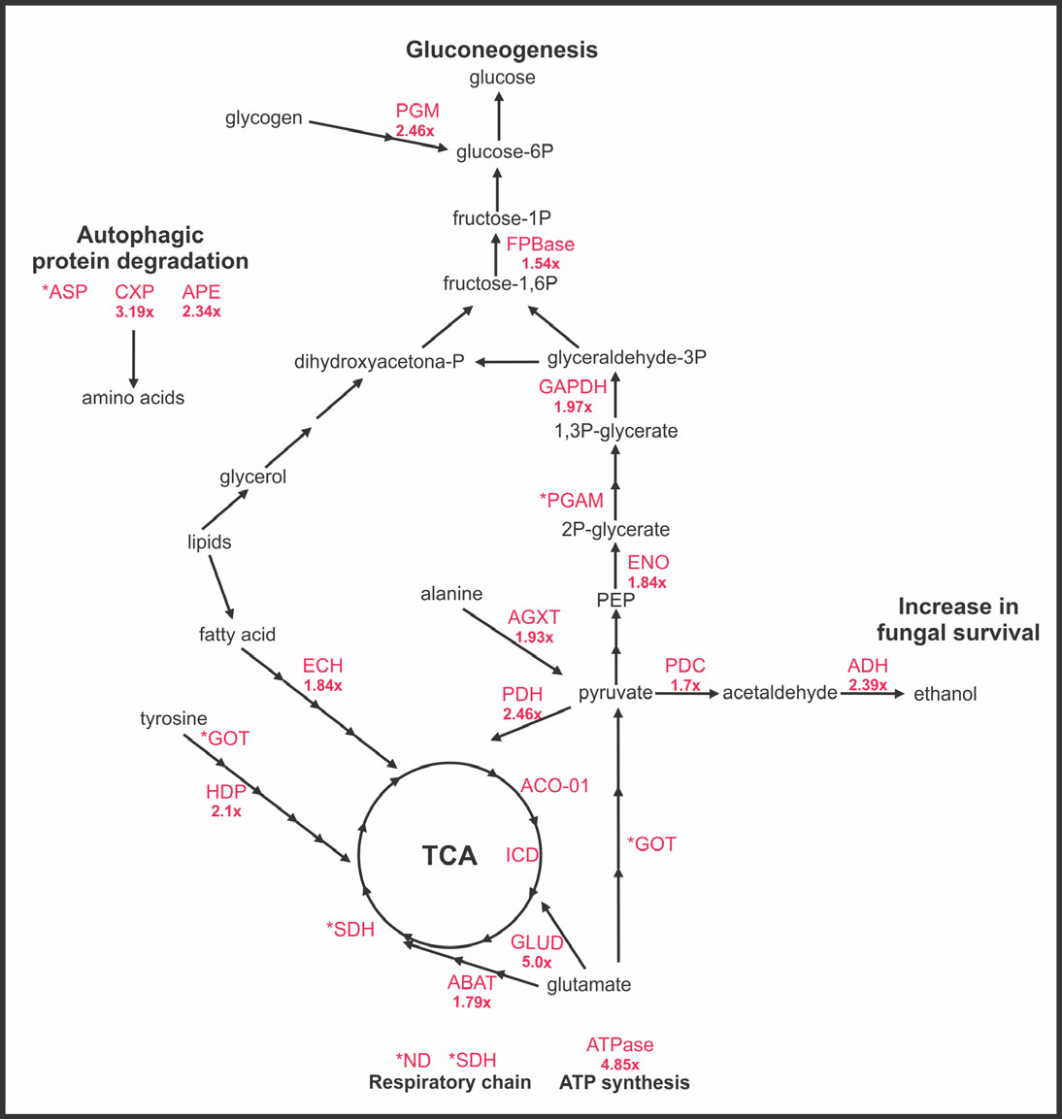
Molecular mechanism used by *P*. *brasiliensis* to survive inside macrophages. The up regulated enzymes in *P*. *brasiliensis* during macrophage interaction are: PGM: phosphoglucomutase; PBPase: fructose 1,6-biphosphatase; GAPDH glyceraldehyde 3-phosphate dehydrogenase; PGAM: phosphoglycerate mutase; ENO: enolase; PDC: pyruvate decarboxylase; ADH: alcohol dehydrogenase; PDH: pyruvate dehydrogenase SDH: succinate dehydrogenase, ECH: enoyl-CoA hydratase; AGXT: alanine glyoxylate aminotransferase; GOT: aspartate aminotransferase; HDP: 4-hydroxyphenylpyruvate dioxygenase; ABAT: 4-aminobutyrate aminotransferase; GLUD: glutamate dehydrogenase; ASP: aspartyl protease; CXP: carboxypepetidase Y; APE: vacuolar aminopeptidase; ND: NADH ubiquinone oxidoreductase; and ATPase: ATP synthase. The numbers before enzyme names represent increasing rates in the protein expression during macrophage interaction. The asterisk represents the proteins detected in *P*. *brasiliensis* only during macrophage infection.

The proteomic approach provided new insights into the molecular mechanisms used by *P*. *brasiliensis* to adapt to the macrophage environment. As depicted in Table 1, fructose1,6-biphosphatase was induced, suggesting an increase in gluconeogenesis to provide glucose. The anaplerotic precursors for glucose are most likely provided by the carbon backbones released by the amino acid degradation pathways (Table 1). Enzymes related to glutamate (glutamate dehydrogenase), alanine (alanine glyoxylate aminotransferase) and aspartate (aspartate aminotransferase) were induced, suggesting their role in production of glucose precursors. Moreover, fatty acids are probably used as fuel for fungal survival inside phagocytes, as suggested by the induction of the enzyme enoyl CoA hydratase. Ethanol production appeared to be increased based on the induction of pyruvate decarboxylase and alcohol dehydrogenase; the pyruvate is most likely provided through amino acid degradation. The increased ethanol production could contribute to fungal survival inside the macrophage because ethanol production contributes to pathogenesis [29]. The up regulated processes induced in *P*. *brasiliensis* upon internalization by macrophages are depicted in Fig 2.

**Table 1:** Selected up-regulated proteins in *P*. *brasiliensis* yeast cells during macrophage infection that are related to alternative carbon metabolism.

Accession number^1^	Protein description	Score^2^	Fold change^3^
**Gluconeogenesis**			
PADG_01706	Fructose 1,6 bisphosphatase	5195.62	1.54
PADG_04059	Enolase	60972.12	1.84
PADG_02411	Glyceraldehyde 3 phosphate dehydrogenase	72801	1.97
PADG_06358	Phosphoglycerate mutase family protein	569.7	*
**Anaerobic metabolism**			
PADG_02271	Alcohol dehydrogenase	874.63	2.39
PADG_00714	Pyruvate decarboxylase	1628.1	1.70
**Glycogen metabolism**			
PADG_00681	Phosphoglucomutase	2716.46	2.46
**Tricarboxilic acid cycle**			
PADG_06494	Dihydrolipoyl dehydrogenase	9770.13	2.46
PADG_00052	Succinate dehydrogenase flavoprotein subunit	1120.04	1.60
PADG_07475	Succinate dehydrogenase flavoprotein subunit	120.14	*
PADG_08013	Succinate dehydrogenase iron sulfur subunit	665.89	*
**Beta-oxidation of fatty acid**			
PADG_01209	Enoyl CoA hydratase	11615.45	1.84
**Amino acid degradation**			
PADG_03020	Alanine glyoxylate aminotransferase	1597.6	1.93
PADG_01621	Aspartate aminotransferase	768.6	*
PADG_08468	4-hydroxyphenylpyruvate dioxygenase	9595.33	2.10
PADG_02214	4-aminobutyrate aminotransferase	1086.24	1.79
PADG_04516	NADP specific glutamate dehydrogenase	1485.79	5.00

^1^Accession number obtained in the *Paracoccidioides* database available at http://www.broadinstitute.org/annotation/genome/paracoccidioides_brasiliensis/MultiHome.html.

^2^PLGS score is the result of different mathematical models for peptide and fragment assignment prediction.

^3^Fold-change values were obtained by dividing the values of protein abundance (in fmol) from *P*. *brasiliensis* yeast cells during macrophage infection by the abundance in the control. Proteins with a minimum fold-change of 50% were considered to be regulated.

* Proteins detected in *P*. *brasiliensis Pb*18 only during macrophage infection.

As depicted in Table 2, the glycolytic-specific enzymes phosphofructokinase 1 and hexokinase were repressed, indicating that glucose was not used as an energy source and reinforcing that *P*. *brasiliensis* was facing a glucose-poor environment inside the macrophages. Quantification of the transcript encoding fructose 1,6 biphosphatase (*pbpase*) corroborated the proteomics data (S7 File.). The inhibition of protein synthesis in the poor environment inside the macrophages was strongly suggested by the down regulation of a large number of proteins related to that process (S2 File and Table 2).

**Table 2:** Selected down-regulated proteins in *P*. *brasiliensis Pb*18 yeast cells during macrophage infection.

Accession number^1^	Protein description	Score^2^	Fold change^3^
**Glycolysis**			
PADG_01896	Phosphoglycerate kinase	10933.7	0.20
PADG_03813	Hexokinase	1005.71	*
PADG_00192	6-phosphofructokinase	1158.94	*
PADG_00668	Fructose bisphosphate aldolase	19990.67	0.37
**Protein synthesis**			
PADG_04057	Translation initiation factor eIF3	4000.27	*
PADG_00932	Translation initiation factor eIF3	478.38	*
PADG_01891	Translation initiation factor RLI1	656.19	*
PADG_06110	Translation factor SUI1	3845.67	*
PADG_00692	Elongation factor 1 alpha	25477.84	0.23
PADG_02759	Ribosome recycling factor domain-containing protein	1902.46	*
PADG_02752	116 kDa U5 small nuclear ribonucleoprotein component	110.37	0.53
PADG_04730	Nascent polypeptide associated complex subunit alpha	1887.05	*
PADG_02896	Elongation factor 1 beta	27699.38	0.36
PADG_06265	Elongation factor 1 gamma 1	21636.54	0.41
PADG_08125	Elongation factor 2	8384.21	0.45
PADG_03431	Putative tRNA-binding protein	533.64	*
PADG_03440	Prolyl tRNA synthetase	454.75	*
PADG_01558	Histidyl tRNA synthetase	1005.73	*
PADG_02484	Valyl tRNA synthetase	844.82	*
PADG_03689	Tyrosyl tRNA synthetase	1451.04	*
PADG_05848	Glycyl tRNA synthetase	600.14	*
PADG_05897	Seryl tRNA synthetase	2707.68	*
PADG_08472	Lysyl tRNA synthetase	857.74	*
PADG_04962	Aspartyl tRNA synthetase	2972.06	0.54
PADG_00785	Ribosomal protein S15	1042.95	*
PADG_01503	37S ribosomal protein Rsm24	553.96	*
PADG_04866	40S ribosomal protein S10 A	3053.9	*
PADG_02445	40S ribosomal protein S15	10623.49	0.39
PADG_06502	40S ribosomal protein S20	7199.8	0.48
PADG_06599	40S ribosomal protein S25	539.48	*
PADG_08605	40S ribosomal protein S28	4991.9	*
PADG_04848	60S ribosomal protein L8 B	14038.4	0.63
PADG_02828	60S ribosomal protein L10a	1216.32	0.59
PADG_07803	60S ribosomal protein L12	9790.37	0.63
PADG_06726	60S ribosomal protein L17	2919.98	0.66
PADG_01026	60S ribosomal protein L43	7369.92	0.66

^1^ Accession number obtained in the *Paracoccidioides* database available at http://www.broadinstitute.org/annotation/genome/paracoccidioides_brasiliensis/MultiHome.html.

^2^ PLGS score is the result of different mathematical models for peptide and fragment assign prediction.

^3^ Fold-change values were obtained by dividing the values of protein abundance (in fmol) from *P*. *brasiliensis* yeast cells during macrophage infection by the abundance in the control. Proteins with a minimum fold-change of 50% were considered to be regulated.

* Proteins detected in *P*. *brasiliensis Pb*18 only under the control condition.

It is important to highlight the overexpression of proteins related to autophagic protein degradation process, such as vacuolar aminopeptidase, carboxypeptidase Y and aspartyl protease (Table 3). The induction of autophagic protein degradation is important because it provides a non-selective pathway for the bulk turnover of cytoplasmic components, thereby generating amino acids during nutrient starvation [30].

**Table 3:** Up regulated proteins putatively related to cell rescue and defense in *P*. *brasiliensis* yeast cells during macrophage infection.

Accession number^1^	Protein description	Score^2^	Fold change^3^
PADG_01479	Gamma glutamyltranspeptidase	577.9	1.95
PADG_07460	Vacuolar aminopeptidase	693.28	2.34
PADG_06314	Carboxypeptidase Y	504.42	3.19
PADG_00634	Aspartyl protease	452.93	*
PADG_07749	Protoplast secreted protein—Y20	38131.91	1.55
PADG_05183	Mitochondrial monothiol glutaredoxin 5	1304.22	*
PADG_02764	Thioredoxin-like protein	2118.45	2.86
PADG_03161	Thioredoxin	647.52	*
PADG_03163	Mitochondrial cytochrome c peroxidase	6455.72	1.68
PADG_07418	Cu/Zn superoxide dismutase	6827.38	1.77

^1^ Accession number obtained in the *Paracoccidioides* database available at http://www.broadinstitute.org/annotation/genome/paracoccidioides_brasiliensis/MultiHome.html.

^2^ PLGS score is the result of different mathematical models for peptide and fragment assign prediction.

^3^ Fold-change values were obtained by dividing the values of protein abundance (in fmol) from *P*. *brasiliensis* yeast cells during macrophage infection by the abundance in the control. Proteins with a minimum fold-change of 50% were considered to be regulated.

* Proteins detected in *P*. *brasiliensis* only during macrophage infection.

### Putative virulence factors up-regulated in *P*. *brasiliensis* during interaction with macrophages

Several proteins that have been described as virulence factors in pathogenic microorganisms were up-regulated in *P*. *brasiliensis* yeast cells interacting with macrophages (Table 3). Some of these proteins may be involved in the oxidative stress response. For example, we detected the up-regulation of one cytochrome C peroxidase, two thioredoxins, and one superoxide dismutase. The transcript encoding the mitochondrial enzyme cytochrome c peroxidase was up-regulated during the interaction of *P*. *brasiliensis* with macrophage cells; the proteomics results were corroborated by qRT-PCR (S7 File). Mutants of these proteins in fungi, bacteria and parasites present attenuated virulence during infection in mice [31,32,33,34,35].

**Fig 3:**
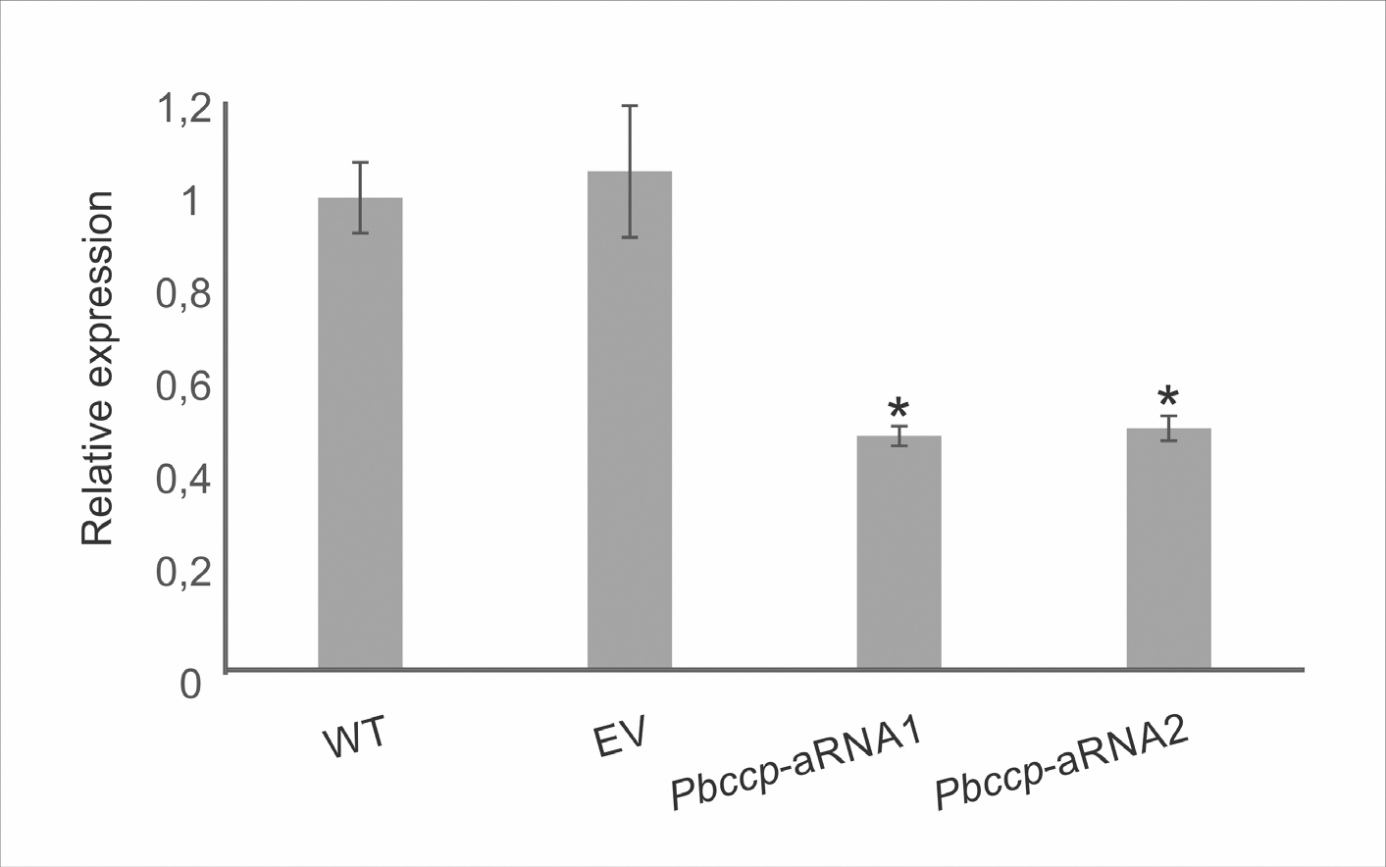
Evaluation of silencing efficiency of cytochrome c peroxidase knockdown mutants (*Pbccp*-aRNA). Relative quantification performed by real-time quantitative PCR to confirm CCP silencing. WT: wild type yeast cells (*Pb*339 strain); EV: yeast cells (*Pb*339 strain) containing the empty vector with no CCP-AS; *Pbccp*-aRNA1 and *Pbccp*-aRNA2: colonies from yeast cells (*Pb*339 strain) containing the cassette with the *ccp* antisense fragment. The Student's t-test was used for statistical comparisons. Error bars represent the standard deviation from three biological replicates, while * represents *p*≤0.05.

**Fig 4:**
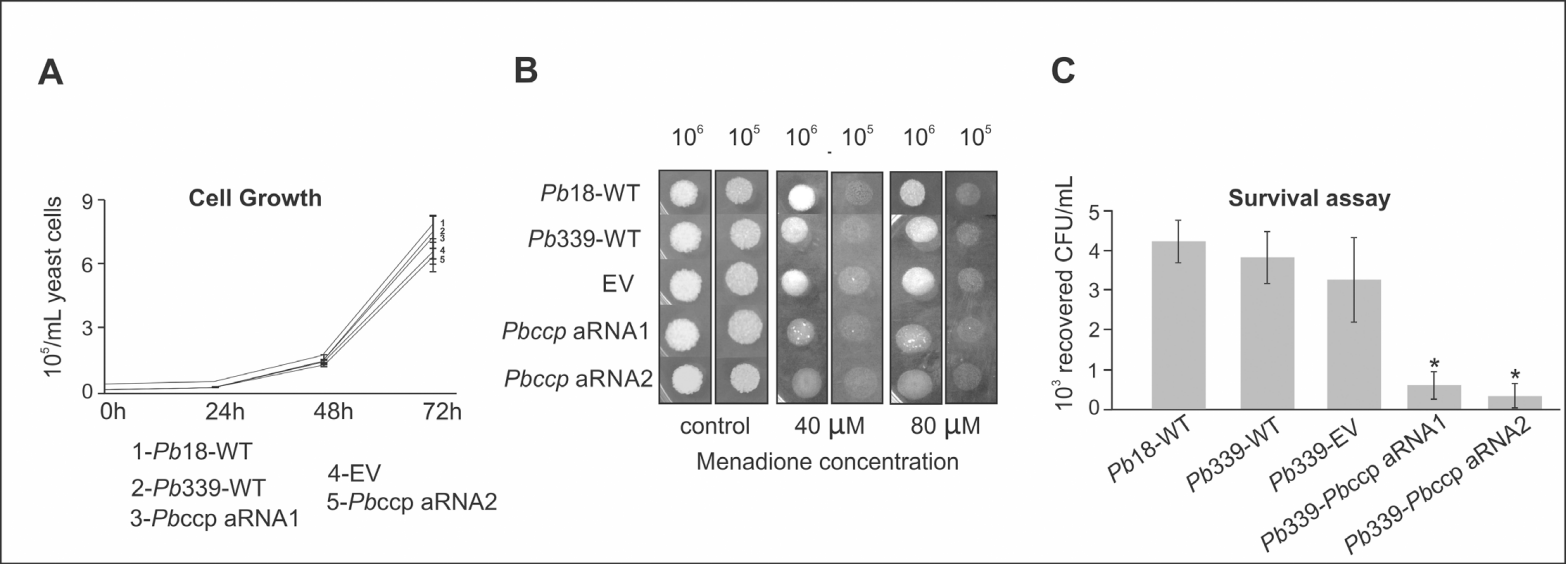
Evaluation of the cell growth, sensitivity of the *Pbccp*-aRNA mutant to oxidative stress and survival in macrophages. **(A)** Curve of cell growth in BHI medium with the strains *Pb*18-WT (wild type *Pb*18 strain), *Pb*339-WT (wild type *Pb*339 strain), EV: yeast cells (*Pb*339 strain containing the empty vector); *Pbccp*-aRNA1 and *Pbccp*-aRNA2: independent colonies of yeast cells (*Pb*339 strain) containing the cassette with the CCP-AS fragment. **(B)**The *Pbccp-*aRNA sensitivity to oxidative stress was examined in the presence of 40 μM and 80 μM of menadione. *P*. *brasiliensis Pb*18-WT, *Pb*339-WT and EV were used as controls. **(C)** Interaction assay of *P*. *brasiliensis* and macrophage cells. The experiments were performed in biological triplicates. *Pb*18-WT: wild type (*Pb*18 strain); *Pb*339-WT: wild type (*Pb*339) EV: yeast cells (*Pb*339 strain) containing the empty vector without CCP-AS; *Pbccp*-aRNA1 and *Pbccp*-aRNA2: independent colonies of yeast cells (*Pb*339 strain) containing the cassette with the CCP-AS fragment. The asterisk denotes *p* < 0.01(Student’s t-test).

Therefore, we analyzed the role of the protein cytochrome c peroxidase (CCP) in fungal virulence because the enzyme contributes to the fungal antioxidant defense [36]. A silenced mutant strain for this gene was generated using antisense technology [15,24,37]. The knockdown mutant was obtained in the *Pb*339 strain, which was demonstrated to be the most feasible strain for genetic transformation in our laboratory [37]. The efficiency of the gene silencing from two *Paracoccidioides* transformants was evaluated by qRT-PCR. The silencing efficiency obtained for the CCP knockdown mutants was approximately 50%. The mutant strain obtained with the empty binary vector (EV) depicted no significant difference in the *ccp* expression level when compared with the wild type strain (WT) (Fig 3). To evaluate the sensitivity of the *ccp* silenced strain to oxidative stress, we analyzed the growth of two *P*. *brasiliensis* isolates (*Pb*18 wild type and *Pb*339 wild type), EV and *Pbccp-*aRNA strains using solid medium supplemented with menadione, that increases mitochondrial-generated ROS stress. The *ccp-*aRNA strains were more sensitive to 40 μM and 80 μM of menadione compared to the wild types isolates and EV strains (Fig 4, Panel B). The strains presented the same *in vitro* growth profile in absence of menadione (Fig 4, panel A). The results strongly suggest that CCP plays a role in avoiding cell damage caused by oxidative stress.

### CCP silencing reduces *P*. *brasiliensis* survival upon macrophage interaction and during infection in BALB/c mice

The survival of two CCP-silenced mutants (*Pbccp-*aRNA1 and *Pbccp*-aRNA2) was assessed during infection of J774 1.6 macrophage cells. The recovery of colony-forming units (CFU) after 24 hours of macrophage infection is depicted in Fig 4, Panel C. The control strains include two isolates of *P*. *brasiliensis* (*Pb*18-WT and *Pb*339-WT) and EV, and showed no significant differences in the number of CFUs recovered from macrophages. In contrast, the number of CFUs recovered from the CCP knockdown strains (*Pbccp*-aRNA1 and *Pbccp*-aRNA2) was severely reduced, suggesting the importance of the CCP protein during *P*. *brasiliensis* phagocytosis by macrophage cells.

**Fig 5:**
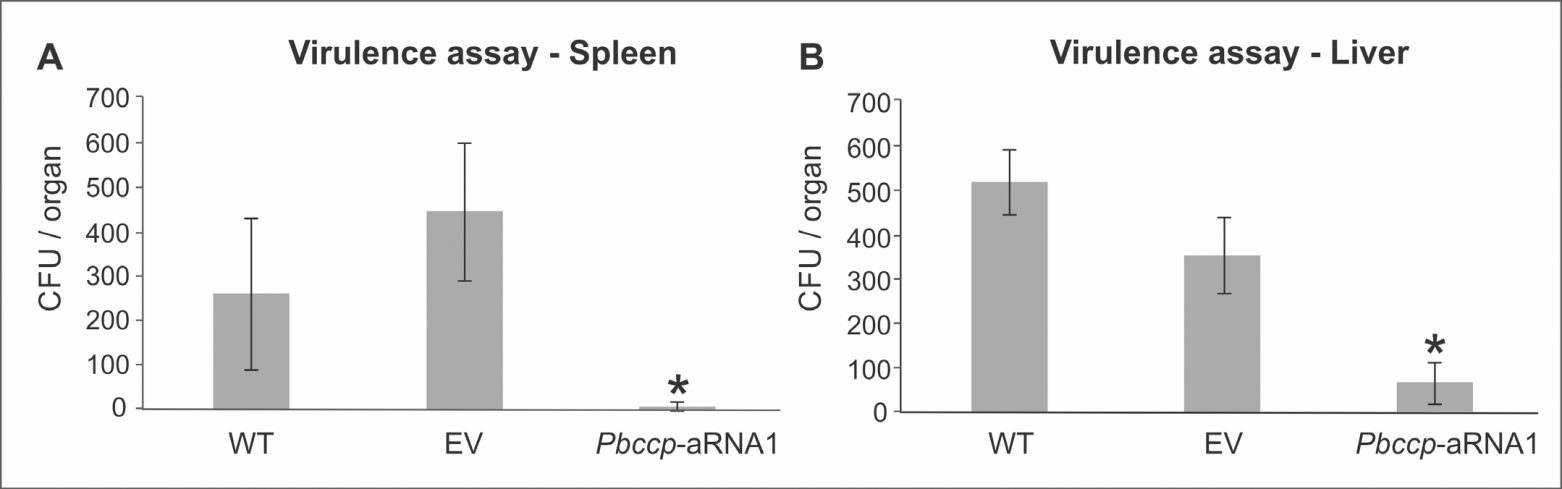
Virulence of the *Pb*ccp-aRNA mutant in the liver and spleen of infected BALB/c mice. Colony forming units recovered from the spleen (A) and liver (B) of mice infected with *P*. *brasiliensis* wild type (WT), *P*. *brasiliensis* containing the empty vector (EV) and the silenced mutant *Pb*ccp-aRNA1. The experiments were performed in biological triplicates. Error bars represent the standard deviation from biological replicates, while * represents *p*-values < 0.01.

To investigate the effect of the CCP gene on *P*. *brasiliensis* during infection, we infected BALB/c mice with the *Pb*ccp-aRNA1 strain and compared the results to mice infected with the WT and EV strains. A strong decrease in fungal survival was observed in the livers and spleens of mice infected with the silenced strain (Fig 5). In contrast, no significant differences in fungal burdens in the spleens and livers were detected in mice infected with the WT and EV strains. The results suggest that CCP is important for the establishment of infection by *P*. *brasiliensis*.

## Discussion

In this work, for the first time, we performed a proteomic analysis of the response of *P*. *brasiliensis* upon interaction with macrophages. A total of 308 proteins were identified as up- or down-regulated in *P*. *brasiliensis* upon macrophage interaction. This number corresponds to proteins detected in at least two of three biological replicates, and presenting at least ± 50% differences in abundance.

The metabolic changes detected in *P*. *brasiliensis* reflect how the yeast cells sense the hostile environment in macrophages. Upon phagocytosis by macrophages, *P*. *brasiliensis* activates responses related to the synthesis of glucose by gluconeogenesis, amino acid catabolism rendering precursors of glucose, and the utilization of fatty acids by beta-oxidation. Additionally, we observed the induction of proteins and enzymes related to ROS detoxification. The results indicated that the anaplerotic precursors for glucose are most likely provided by the carbon backbones released from the amino acid degradation pathways. Enzymes related to glutamate (glutamate dehydrogenase), alanine (alanine glyoxylate aminotransferase) and aspartate (aspartate aminotransferase) catabolism were induced, indicating a possible increase in these catabolic pathways that may result in an enrichment of glucose precursors such as pyruvate, fumarate and oxaloacetate. Ethanol production could be increased, based on the induction of pyruvate decarboxylase and alcohol dehydrogenase and could lead to fungal survival inside the macrophage because ethanol production contributes to pathogenesis [29]. In this way, *P*. *brasiliensis* might remodel its metabolism to recycle its own carbon-containing molecules. The data suggest that gluconeogenesis play an important role in the adaptive responses to phagocytosis. The induction of gluconeogenesis and amino acid degradation enzymes suggest that *P*. *brasiliensis* can use carbon backbones of amino acids to synthesize glucose, presumably from the host.

Phagocytic cells generate ROS to eliminate fungi, which are very efficient pathogens in responding to oxidative stress [12]. A number of proteins and transcripts were involved in antioxidant defense systems have been described in *P*. *brasiliensis in vitro* and upon macrophage phagocytosis [14,17]. The fungus induces the accumulation of detoxifying enzymes such as superoxide dismutases, cytochrome c peroxidase and thioredoxins in response to H_2_O_2_ [14]. Of special note, in this work we demonstrated that the cytochrome c peroxidase (CCP) plays a role in the *P*. *brasiliensis* response to oxidative stress during interaction with macrophage cells and infection in a murine model. We previously demonstrated that CCP promotes *Paracoccidioides* sp. protection against nitrosative stress, *in vitro*, as demonstrated by the sensitivity of the *Pb*ccp-aRNA1 strain to S-nitrosoglutathione, (GSNO), indicating an interface of the role played by CCP in oxidative and nitrosative stress responses [15]. The CCP protein is related to the oxidative stress response in other fungi, such as *Cryptococcus neoformans*. A CCP mutant in *C*. *neoformans* presented a reduction in intracellular growth when cultured with macrophages [36]. Based on our results, cytochrome c peroxidase can be considered a virulence factor because protein silencing promoted a decrease in the number of recovered fungi in macrophages and in an animal model.

Other potential virulence factors induced during macrophage infection were detected in our analysis in *P*. *brasiliensis* following macrophage infection.

Autophagy is presumably induced in *P*. *brasiliensis* upon macrophage infection. Autophagy is a vacuolar trafficking pathway that targets subcellular constituents to the vacuole for degradation and recycling; carboxypeptidase Y, vacuolar aminopeptidases and aspartyl protease are involved in this process [38]. The up-regulation of proteins, described as virulence factor in other pathogens, during *P*. *brasiliensis* interaction with macrophages suggests that these proteins may be important during infection of the human host by *P*. *brasiliensis*.

Taken together our results show that *P*. *brasiliensis* responds to several stress conditions once inside macrophages, including glucose deprivation and oxidative stress. The fungal response to glucose deprivation includes a metabolic shift from glycolysis to gluconeogenesis in which glucose precursors are provided by the catabolism of amino acids, as demonstrated by our proteomic analysis. We also assessed the contribution of the fungal oxidative stress response mediated by cytochrome c peroxidase for the survival of *P*. *brasiliensis* using knockdown strains. According to our data, the enzyme plays a relevant role in fungal survival inside macrophages, and therefore can be described as a virulence factor.

## Supporting Information

S1 File Up-regulated proteins of *P*. *brasiliensis* during macrophage infection in J774 1.6 cells. (DOCX)

S2 File Down-regulated proteins of *P*. *brasiliensis* during macrophage infection in J774 1.6 cells. (DOCX)

S3 File NanoUPLC-MSE data quality analysis. PepFrag1 and PepFrag2 correspond to the peptides matches compared to the database by PLGS, VarMod corresponds to variable modifications, In Source corresponds to fragmentation that occurred in the ionization source, Missed Cleavage indicates the missed cleavage performed by trypsin and Neutral loss HO and NH correspond to water and ammonia precursor losses.
(PDF)

S4 File Mass error of the identified fragments. The number of identified fragments according to the error range (x-axis).
(PDF)

S5 File Detection dynamic range. Quantified fragments were sorted according to the fragment amount (Fmol) and plotted in the graphics as grey circles. Standard protein was indicated by red circle. A protein with a low coefficient of variance between samples was used to normalize the expression data and allow comparisons of the control and *P*. *brasiliensis* data from infected macrophage.
(PDF)

S6 File Functional categorization of *P*. *brasiliensis*-regulated proteins during macrophage infection. (A) Abundance (%) of upregulated *P*. *brasiliensis* proteins during the interaction with macrophages in agreement with their biological functions. (B) Abundance (%) of down-regulated *P*. *brasiliensis* proteins during the interaction with macrophages in agreement with their biological functions.
(PDF)

S7 File Quantification of transcripts encoding proteins that were up-regulated during macrophage infection. Transcript levels of genes encoding fructose 1,6 biphosphatase (pbase) and cytochrome c peroxidase (ccp). Transcript levels were measured using quantitative RT-PCR. Data were normalized to the beta tubulin protein transcript and are presented as fold change calculated based on the rate of macrophage interaction to control condition. The Student's t-test was used for statistical comparisons. Error bars represent the standard deviation from three biological replicates, while * represents p≤0.05.
(PDF)

## References

[1] San-Blas G, Nino-Vega G, Iturriaga T (2002) *Paracoccidioides brasiliensis* and paracoccidioidomycosis: molecular approaches to morphogenesis, diagnosis, epidemiology, taxonomy and genetics. Med Mycol 40: 225–242. 12146752 10.1080/mmy.40.3.225.242

[2] Brummer E, Castaneda E, Restrepo A (1993) Paracoccidioidomycosis: an update. Clin Microbiol Rev 6: 89–117. 8472249 10.1128/cmr.6.2.89 PMC358272

[3] McEwen JG, Brummer E, Stevens DA, Restrepo A (1987) Effect of murine polymorphonuclear leukocytes on the yeast form of *Paracoccidioides brasiliensis* . Am J Trop Med Hyg 36: 603–608. 3555139 10.4269/ajtmh.1987.36.603

[4] Lacaz CS (1994) Historical evolution of the knowledge on paracoccidioidomycosis and its etiological agent, *Paracoccidioides brasiliensis* In: Franco M, Lacaz CS, Restrepo-Moreno A, del Negro GB, editors. Paracoccidioidomycosis. London: CRC Press pp. 1–7.

[5] Brummer E, Hanson LH, Restrepo A, Stevens DA (1988) In vivo and in vitro activation of pulmonary macrophages by IFN-gamma for enhanced killing of *Paracoccidioides brasiliensis* or *Blastomyces dermatitidis* . J Immunol 140: 2786–2789. 3128610

[6] Brummer E, Hanson LH, Stevens DA (1988) Gamma-interferon activation of macrophages for killing of *Paracoccidioides brasiliensis* and evidence for nonoxidative mechanisms. Int J Immunopharmacol 10: 945–952. 3145925 10.1016/0192-0561(88)90041-0

[7] Brummer E, Hanson LH, Restrepo A, Stevens DA (1989) Intracellular multiplication of *Paracoccidioides brasiliensis* in macrophages: killing and restriction of multiplication by activated macrophages. Infect Immun 57: 2289–2294. 2744848 10.1128/iai.57.8.2289-2294.1989 PMC313444

[8] Moscardi-Bacchi M, Brummer E, Stevens DA (1994) Support of *Paracoccidioides brasiliensis* multiplication by human monocytes or macrophages: inhibition by activated phagocytes. J Med Microbiol 40: 159–164. 8114064 10.1099/00222615-40-3-159

[9] Fang FC (2004) Antimicrobial reactive oxygen and nitrogen species: concepts and controversies. Nat Rev Microbiol 2: 820–832. 15378046 10.1038/nrmicro1004

[10] Hampton MB, Kettle AJ, Winterbourn CC (1998) Inside the neutrophil phagosome: oxidants, myeloperoxidase, and bacterial killing. Blood 92: 3007–3017. 9787133

[11] Ferrari CK, Souto PC, Franca EL, Honorio-Franca AC (2011) Oxidative and nitrosative stress on phagocytes' function: from effective defense to immunity evasion mechanisms. Arch Immunol Ther Exp (Warsz) 59: 441–448. 21972015 10.1007/s00005-011-0144-z

[12] Brown AJ, Haynes K, Quinn J (2009) Nitrosative and oxidative stress responses in fungal pathogenicity. Curr Opin Microbiol 12: 384–391. 10.1016/j.mib.2009.06.007 19616469 PMC2728829

[13] Campos EG, Jesuino RS, Dantas Ada S, Brigido Mde M, Felipe MS (2005) Oxidative stress response in Paracoccidioides brasiliensis. Genet Mol Res 4: 409–429. 16110454

[14] Grossklaus DA, Bailao AM, Vieira Rezende TC, Borges CL, de Oliveira MA, Parente JA, et al (2013) Response to oxidative stress in *Paracoccidioides* yeast cells as determined by proteomic analysis. Microbes Infect 15: 347–364. 10.1016/j.micinf.2012.12.002 23421979

[15] Parente AF, Naves PE, Pigosso LL, Casaletti L, McEwen JG, Parente-Rocha JA, et al (2015) The response of *Paracoccidioides* spp. to nitrosative stress. Microbes Infect 17: 575–585. 10.1016/j.micinf.2015.03.012 25841799

[16] Lima PS, Casaletti L, Bailao AM, Vasconcelos AT, Fernandes GR, Soares CMA (2014) Transcriptional and proteomic responses to carbon starvation in *Paracoccidioides* . PLoS Negl Trop Dis 8: e2855 10.1371/journal.pntd.0002855 24811072 PMC4014450

[17] Tavares AH, Silva SS, Dantas A, Campos EG, Andrade RV, Maranhao AQ, et al (2007) Early transcriptional response of *Paracoccidioides brasiliensis* upon internalization by murine macrophages. Microbes Infect 9: 583–590. 17387029 10.1016/j.micinf.2007.01.024

[18] Matute DR, McEwen JG, Puccia R, Montes BA, San-Blas G, Bagagli E, et al (2006) Cryptic speciation and recombination in the fungus *Paracoccidioides brasiliensis* as revealed by gene genealogies. Mol Biol Evol 23: 65–73. 16151188 10.1093/molbev/msj008

[19] Matute DR, Sepulveda VE, Quesada LM, Goldman GH, Taylor JW, Restrepo A, et al (2006) Microsatellite analysis of three phylogenetic species of *Paracoccidioides brasiliensis* . J Clin Microbiol 44: 2153–2157. 16757613 10.1128/JCM.02540-05 PMC1489427

[20] da Fonseca CA, Jesuino RS, Felipe MS, Cunha DA, Brito WA, Soares CM (2001) Two-dimensional electrophoresis and characterization of antigens from Paracoccidioides brasiliensis. Microbes Infect 3: 535–542. 11418327 10.1016/s1286-4579(01)01409-5

[21] Murad AM, Rech EL (2012) NanoUPLC-MSE proteomic data assessment of soybean seeds using the Uniprot database. BMC Biotechnol 12: 82 10.1186/1472-6750-12-82 23126227 PMC3532185

[22] Murad AM, Souza GH, Garcia JS, Rech EL (2011) Detection and expression analysis of recombinant proteins in plant-derived complex mixtures using nanoUPLC-MS(E). J Sep Sci 34: 2618–2630. 10.1002/jssc.201100238 21898799

[23] Geromanos SJ, Vissers JP, Silva JC, Dorschel CA, Li GZ, Gorenstein MV, et al (2009) The detection, correlation, and comparison of peptide precursor and product ions from data independent LC-MS with data dependant LC-MS/MS. Proteomics 9: 1683–1695. 10.1002/pmic.200800562 19294628

[24] Menino JF, Almeida AJ, Rodrigues F (2012) Gene knockdown in *Paracoccidioides brasiliensis* using antisense RNA. Methods Mol Biol 845: 187–198. 10.1007/978-1-61779-539-8_12 22328375

[25] Sambrook J, Russel DW (2001) Molecular Cloning. A Laboratory Manual. New York: Cold Spring Harbor Laboratory Press.

[26] Bookout AL, Cummins CL, Mangelsdorf DJ, Pesola JM, Kramer MF (2006) High-throughput real-time quantitative reverse transcription PCR. Curr Protoc Mol Biol Chapter 15: Unit 15 18. 10.1002/0471142727.mb1508s73 18265376

[27] Bailão AM, Schrank A, Borges CL, Dutra V, Molinari-Madlum EEWI, Felipe MSS, et al (2006) Differential gene expression by *Paracoccidioides brasiliensis* in host interaction conditions: representational difference analysis identifies candidate genes associated with fungal pathogenesis. Microbes Infect 8: 2686–2697. 16962356 10.1016/j.micinf.2006.07.019

[28] Steinberg BE, Touret N, Vargas-Caballero M, Grinstein S (2007) In situ measurement of the electrical potential across the phagosomal membrane using FRET and its contribution to the proton-motive force. Proc Natl Acad Sci U S A 104: 9523–9528. 17517624 10.1073/pnas.0700783104 PMC1890527

[29] Grahl N, Puttikamonkul S, Macdonald JM, Gamcsik MP, Ngo LY, Hohl TM, et al (2011) In vivo hypoxia and a fungal alcohol dehydrogenase influence the pathogenesis of invasive pulmonary aspergillosis. PLoS Pathog 7: e1002145 10.1371/journal.ppat.1002145 21811407 PMC3141044

[30] Takeshige K, Baba M, Tsuboi S, Noda T, Ohsumi Y (1992) Autophagy in yeast demonstrated with proteinase-deficient mutants and conditions for its induction. J Cell Biol 119: 301–311. 1400575 10.1083/jcb.119.2.301 PMC2289660

[31] Schmidt M, Klimentova J, Rehulka P, Straskova A, Spidlova P, Szotakova B, et al (2013) *Francisella tularensis* subsp. holarctica DsbA homologue: a thioredoxin-like protein with chaperone function. Microbiology 159: 2364–2374. 10.1099/mic.0.070516-0 24014665

[32] Shi Y, Liu L, Zhang T, Shen L, Zhang J, Zhang Y, et al (2013) The involvement of *Helicobacter pylori* thioredoxin-1 in gastric carcinogenesis. J Med Microbiol 62: 1226–1234. 10.1099/jmm.0.056903-0 23558136

[33] Matthews K, Kalanon M, Chisholm SA, Sturm A, Goodman CD, Dixon MW, et al (2013) The Plasmodium translocon of exported proteins (PTEX) component thioredoxin-2 is important for maintaining normal blood-stage growth. Mol Microbiol 89: 1167–1186. 10.1111/mmi.12334 23869529

[34] Martin DW, Baumgartner JE, Gee JM, Anderson ES, Roop RM 2nd (2012) SodA is a major metabolic antioxidant in *Brucella abortus* 2308 that plays a significant, but limited, role in the virulence of this strain in the mouse model. Microbiology 158: 1767–1774. 10.1099/mic.0.059584-0 22556360 PMC3542146

[35] Xu L, Chen W (2013) Random T-DNA mutagenesis identifies a Cu/Zn superoxide dismutase gene as a virulence factor of *Sclerotinia sclerotiorum* . Mol Plant Microbe Interact 26: 431–441. 10.1094/MPMI-07-12-0177-R 23252459

[36] Giles SS, Perfect JR, Cox GM (2005) Cytochrome c peroxidase contributes to the antioxidant defense of *Cryptococcus neoformans* . Fungal Genet Biol 42: 20–29. 15588993 10.1016/j.fgb.2004.09.003

[37] Bailao EF, Parente JA, Pigosso LL, Castro KP, Fonseca FL, Silva-Bailao MG, et al (2014) Hemoglobin Uptake by *Paracoccidioides* spp. Is Receptor-Mediated. PLoS Negl Trop Dis 8: e2856 10.1371/journal.pntd.0002856 24831516 PMC4022528

[38] Khalfan WA, Klionsky DJ (2002) Molecular machinery required for autophagy and the cytoplasm to vacuole targeting (Cvt) pathway in *Saccharomyces cerevisiae* . Curr Opin Cell Biol 14: 468–475. 12383798 10.1016/s0955-0674(02)00343-5

