# Supplementary material for: Macrophage Interaction with Paracoccidioides brasiliensis Yeast Cells Modulates Fungal Metabolism and Generates a Response to Oxidative Stress
Source: PLoS One. 2015 Sep 11;10(9):e0137619. doi: 10.1371/journal.pone.0137619 (PMC4567264; doi:10.1371/journal.pone.0137619)
Supplement: S1 File — (DOCX) [file pone.0137619.s001.docx]

**Supplementary table 1 –** Up-regulated proteins of *P. brasiliensis* during macrophage infection in J774 cells.

| **Accession number^1^** | **Protein description** | **Score^2^** | **Fold change^3^** | **p value^4^** |
| --- | --- | --- | --- | --- |
| **Amino acid metabolism** | |  |  |  |
| PADG_02214 | 4-aminobutyrate aminotransferase | 1086.24 | 1.79 | 1 |
| PADG_04570 | Branched chain amino acid aminotransferase | 572.97 | 1.80 | 1 |
| PADG_01621 | Aspartate aminotransferase | 768.6 | * | * |
| PADG_04516 | NADP specific glutamate dehydrogenase | 1485.79 | 5.00 | 1 |
| PADG_05085 | Delta 1 pyrroline 5 carboxylate dehydrogenase | 3968.2 | 1.57 | 1 |
| PADG_00637 | Arginase | 458.37 | 4.31 | 1 |
| PADG_02777 | Threonine synthase | 595.36 | * | * |
| PADG_06252 | 1,2-dihydroxy 3 keto 5 methylthiopentene dioxygenase | 754.99 | * | * |
| PADG_03020 | Alanine glyoxylate aminotransferase | 1597.6 | 1.93 | 1 |
| PADG_08468 | 4-hydroxyphenylpyruvate dioxygenase | 9595.33 | 2.10 | 1 |
| PADG_07907 | Acetolactate synthase | 1269.3 | 2.14 | 1 |
| PADG_04854 | Anthranilate synthase component | 349.36 | 1.58 | 1 |
| PADG_06671 | 3-isopropylmalate dehydrogenase A | 525.06 | 3.53 | 1 |
| PADG_03114 | Phospho 2 dehydro 3 deoxyheptonate aldolase | 862.15 | * | * |
| **Nitrogen and sulfur metabolismo** | |  |  |  |
| PADG_06490 | Formamidase | 4525.27 | * | * |
| **Nucleotide and nucleoside metabolismo** | |  |  |  |
| PADG_02183 | ADP ribose pyrophosphatase | 2107.17 | * | * |
| PADG_08066 | Purine nucleoside phosphorylase | 1018.83 | * | * |
| PADG_05225 | Orotidine 5 phosphate decarboxylase | 1913.43 | * | * |
| PADG_00331 | Uricase | 746.21 | * | * |
| PADG_04828 | Adenylosuccinate lyase | 2431.85 | 2.34 | 1 |
| PADG_02561 | Atpase alpha subunit | 13537.11 | 2.69 | 1 |
| **Signal transduction** | |  |  |  |
| PADG_06273 | Calcineurin subunit B | 976.11 | * | * |
| PADG_02017 | Calmodulin | 2829.23 | 4.10 | 1 |
| PADG_00282 | GTP binding protein SAS1 | 1656.09 | * | * |
| PADG_03522 | Phosphorylase family protein | 2028.21 | 3.32 | 1 |
| **DNA repair** | |  |  |  |
| PADG_02683 | DNA repair protein RAD23 | 1882.35 | 1.86 | 1 |
| **C-comppound and carbohydrate metabolism** | |  |  |  |
| PADG_04939 | Succinyl coa 3 ketoacid coenzyme A transferase subunit B | 1823.47 | 2.25 | 1 |
| PADG_05855 | Lactonohydrolase | 684.03 | 2.39 | 1 |
| PADG_00912 | UDP galactopyranose mutase | 3906.35 | 2.29 | 1 |
| PADG_07615 | Glucan 1 3 beta glucosidase | 660.12 | * | * |
| **Pentose phosphate pathway** | |  |  |  |
| PADG_07771 | 6 phosphogluconolactonase | 1595.8 | 1.84 | 1 |
| **Glycolysis** | |  |  |  |
| PADG_04059 | Enolase | 60972.12 | 1.84 | 1 |
| PADG_02411 | Glyceraldehyde 3 phosphate dehydrogenase | 72801 | 1.97 | 1 |
| PADG_06358 | Phosphoglycerate mutase family protein | 569.7 | * | * |
| **Gluconeogenesis** | |  |  |  |
| PADG_01706 | Fructose 1 6 bisphosphatase | 5195.62 | 1.54 | 1 |
| **Anaerobic metabolismo** | |  |  |  |
| PADG_02271 | Alcohol dehydrogenase | 874.63 | 2.39 | 1 |
| PADG_00714 | Pyruvate decarboxylase | 1628.1 | 1.70 | 1 |
| **Tricarboxylic acid cycle** | |  |  |  |
| PADG_06494 | Dihydrolipoyl dehydrogenase | 9770.13 | 2.46 | 1 |
| PADG_00052 | Succinate dehydrogenase flavoprotein subunit | 1120.04 | 1.60 | 1 |
| PADG_07475 | Succinate dehydrogenase flavoprotein subunit | 120.14 | * | * |
| PADG_08013 | Succinate dehydrogenase iron sulfur subunit | 665.89 | * | * |
| **Glyoxylate cycle** | |  |  |  |
| PADG_01483 | Isocitrate lyase | 2004.06 | 1.63 | 1 |
| **Glycogen catabolism** | |  |  |  |
| PADG_00681 | Phosphoglucomutase | 2716.46 | 2.46 | 1 |
| **Electron transport and respiration** | |  |  |  |
| PADG_03559 | Cytochrome b5 | 2557.41 | * | * |
| PADG_01366 | NADH-ubiquinone oxidoreductase | 3474.65 | * | * |
| PADG_02745 | NADH ubiquinone oxidoreductase | 1333.29 | * | * |
| **ATP synthesis** | |  |  |  |
| PADG_00688 | Mitochondrial F1F0 ATP synthase subunit Atp14 | 5922.63 | * | * |
| PADG_04729 | ATP synthase D chain mitochondrial | 826.29 | 2.34 | 1 |
| PADG_07813 | ATP synthase gamma chain | 1496.76 | 2.01 | 1 |
| PADG_08349 | ATP synthase subunit beta | 25936.79 | 2.66 | 1 |
| PADG_07789 | ATP synthase delta chain | 1261.26 | 4.85 | 1 |
| **Beta-oxidation of fatty acid** | |  |  |  |
| PADG_01209 | Enoyl coa hydratase | 11615.45 | 1.84 | 1 |
| **Lipid metabolismo** | |  |  |  |
| PADG_01363 | Acyl coa binding protein | 3006.95 | 1.95 | 1 |
| PADG_06382 | Acetyl coa acetyltransferase | 4214.67 | 3.19 | 1 |
| PADG_05310 | Leukotriene A hydrolase | 692 | * | * |
| PADG_07699 | S formylglutathione hydrolase | 2590.55 | * | * |
| PADG_08018 | Glycerol 3 phosphate dehydrogenase | 594.46 | * | * |
| **Biosynthesis of vitamins and cofactors** | |  |  |  |
| PADG_05822 | Pyridoxine biosynthesis protein PDX1 | 8184.32 | 1.84 | 1 |
| PADG_01886 | Adenosylhomocysteinase | 29827.71 | 2.39 | 1 |
| PADG_05474 | Thiamine pyrophosphokinase | 477.33 | 3.25 | 1 |
| PADG_00607 | Riboflavin synthase alpha chain | 1575.84 | * | * |
| PADG_04603 | Spermidine synthase | 2288.22 | 1.77 | 1 |
| PADG_08108 | Coproporphyrinogen III oxidase | 1670.3 | * | * |
| PADG_08328 | Cobalamin independent synthase | 7405.12 | 2.20 | 1 |
| **Mitotic cell and cell cycle control** | |  |  |  |
| PADG_03905 | Proliferating cell nuclear antigen | 7967.5 | 3.49 | 1 |
| PADG_05615 | Ran specific gtpase activating protein | 13103.23 | 1.67 | 1 |
| PADG_03073 | Nuclear movement protein nudc | 4613.94 | 1.84 | 1 |
| **Cell growth and morphogenesis** | |  |  |  |
| PADG_08615 | Tropomyosin | 10023.92 | 3.32 | 1 |
| PADG_06691 | Alpha 1,4 amylase | 532.98 | * | * |
| PADG_08350 | 1,4 alpha glucan branching enzyme | 598.52 | 4.48 | 1 |
| **Transcriptional control** | |  |  |  |
| PADG_00872 | Histone H4 | 5533.47 | * | * |
| PADG_00873 | Histone H3 | 2942.48 | * | * |
| **Transcription** | |  |  |  |
| PADG_05393 | Mrna decapping hydrolase | 676.79 | 2.89 | 1 |
| PADG_02825 | Ribonucleoprotein LSM domain | 1707.02 | * | * |
| **Protein synthesis** | |  |  |  |
| PADG_04735 | Signal recognition particle protein | 579.28 | * | * |
| PADG_01079 | Translation initiation factor 4B | 450.49 | * | * |
| PADG_04657 | Nascent polypeptide associated complex subunit beta | 954.52 | * | * |
| PADG_01914 | Ribosomal protein L35 | 2949.8 | * | * |
| PADG_07924 | 60S ribosomal protein L24 | 2095.76 | * | * |
| PADG_00046 | 60s ribosomal protein mitochondrial precursor | 1153.31 | * | * |
| PADG_08244 | 60S acidic ribosomal protein P1 | 28689.8 | 3.53 | 1 |
| PADG_02446 | 60S acidic ribosomal protein P2 | 15620.39 | 3.97 | 1 |
| PADG_06249 | Glutaminyl trna synthetase | 956.71 | 2.10 | 1 |
| PADG_04288 | L PSP endoribonuclease family protein Hmf1 | 37089.53 | 2.12 | 1 |
| **Protein folding and stabilization** | |  |  |  |
| PADG_07599 | Peptidylprolyl isomerase B | 1623.05 | 1.54 | 1 |
| PADG_01852 | Small glutamine rich tetratricopeptide repeat containing protein | 2516.5 | 2.34 | 1 |
| PADG_00759 | Prefoldin subunit 4 | 2058.89 | * | * |
| **Protein degradation** | |  |  |  |
| PADG_07891 | Ubiquitin | 7292.33 | * | * |
| PADG_06766 | Mitochondrial processing peptidase subunit beta | 686.78 | 1.54 | 1 |
| PADG_05922 | Cytosolic non specific dipeptidase | 4498.78 | 1.70 | 1 |
| PADG_05820 | Xaa Pro aminopeptidase | 619.84 | 2.56 | 1 |
| PADG_05193 | Xaa Pro aminopeptidase | 1579.76 | * | * |
| PADG_02421 | Insulin degrading enzyme | 366.54 | * | * |
| PADG_00615 | Proteasome subunit alpha type 6 | 2488.98 | * | * |
| PADG_04076 | Proteasome component C11 | 626.42 | 1.68 | 1 |
| PADG_08442 | Proteasome component Y13 | 574.28 | 1.58 | 1 |
| PADG_07190 | Proteasome component Y7 | 1806.88 | 1.51 | 1 |
| PADG_03965 | Proteasome component Pre4 | 682.25 | 1.55 | 1 |
| PADG_03982 | Proteasome component C1 | 1025.8 | 1.63 | 1 |
| PADG_03727 | Proteasome component PUP1 | 853.93 | 2.41 | 1 |
| PADG_03680 | Proteasome component PRE2 | 1212.62 | * | * |
| **Cell Rescue, Defense and Virulence** | |  |  |  |
| PADG_01479 | Gamma glutamyltranspeptidase | 577.9 | 1.95 | 1 |
| PADG_07460 | Vacuolar aminopeptidase | 693.28 | 2.34 | 1 |
| PADG_06314 | Carboxypeptidase Y | 504.42 | 3.19 | 1 |
| PADG_00634 | Aspartyl protease | 452.93 | * | * |
| PADG_06992 | Mitochondrial co chaperone grpe | 6890.69 | 2.39 | 1 |
| PADG_07749 | Protoplast secreted protein - Y20 | 38131.91 | 1.55 | 1 |
| PADG_05183 | Mitochondrial monothiol glutaredoxin 5 | 1304.22 | * | * |
| PADG_02764 | Thioredoxin-like protein | 2118.45 | 2.86 | 1 |
| PADG_03161 | Thioredoxin | 647.52 | * | * |
| PADG_03163 | Mitochondrial cytochrome c peroxidase | 6455.72 | 1.68 | 1 |
| PADG_07418 | Cu/Zn superoxide dismutase | 6827.38 | 1.77 | 1 |
| PADG_00430 | Mitochondrial HSP SSC1 | 25098.13 | 1.52 | 1 |
| PADG_04984 | Mitochondrial HSP 10 | 99417.27 | 2.36 | 1 |
| PADG_08369 | Hsp 60 | 63687.71 | 2.86 | 1 |
| **Unclassified** | |  |  |  |
| PADG_07627 | 4-carboxymuconolactone decarboxylase family protein | 614.44 | * | * |
| PADG_04439 | Mitochondrial atpase inhibitor | 16899.15 | 3.67 | 1 |
| PADG_06190 | CCR4 Not complex subunit Caf16 | 571.48 | * | * |
| PADG_03654 | Conserved hypothetical protein | 966.29 | * | * |
| PADG_06087 | Dsdna-binding protein PDCD5 | 1389.93 | * | * |
| PADG_03115 | Hypothetical protein | 2667.55 | 2.10 | 1 |
| PADG_00944 | Methyltransferase domain containing protein | 324.84 | * | * |
| PADG_03886 | Conserved hypothetical protein | 746.11 | * | * |
| PADG_08724 | RPEL repeat protein | 594.42 | * | * |
| PADG_01010 | Predicted protein | 1333.03 | * | * |
| PADG_05584 | Hypothetical protein | 1281.44 | * | * |
| PADG_05703 | Hypothetical protein | 6556.04 | * | * |
| PADG_05739 | NAP family protein | 615.85 | * | * |
| PADG_08736 | Conserved hypothetical protein | 509.84 | * | * |

^1^ Accession number obtained in the *Paracoccidioides* database available at <http://www.broadinstitute.org/annotation/genome/paracoccidioides_brasiliensis/MultiHome.html>.

^2^ PLGS score is the result of different mathematical models for peptide and fragment assign prediction. Acceptable score values consider protein identification with a minimum confidence level of 95% and a false discovery rate of 4%.

^3^ Fold change values were obtained by dividing the values of protein abundance (in fmol) from *P. brasiliensis* yeast cells during macrophage infection by the abundance in control**.** Proteins with a minimum fold change of 50% were considered regulated.

^4^ *p*-value of the fold change. It were considered statistically significant the p-values less than 0.05 and higher than 0.95.

*Proteins detected in *P. brasiliensis* *Pb*18 only during macrophage infection.
