# Supplementary material for: Macrophage Interaction with Paracoccidioides brasiliensis Yeast Cells Modulates Fungal Metabolism and Generates a Response to Oxidative Stress
Source: PLoS One. 2015 Sep 11;10(9):e0137619. doi: 10.1371/journal.pone.0137619 (PMC4567264; doi:10.1371/journal.pone.0137619)
Supplement: S2 File — (DOCX) [file pone.0137619.s002.docx]

**Supplementary table 2–** Down-regulated proteins of *P. brasiliensis* during macrophage infection in J774 cells.

| **Accession number^1^** | **Protein description** | **Score^2^** | **Fold change^3^** | **p value^4^** |
| --- | --- | --- | --- | --- |
| **Aminoacids metabolism** | |  |  |  |
| PADG_00386 | Phospho 2 dehydro 3 deoxyheptonate aldolase | 358.92 | * | * |
| PADG_04698 | Aromatic ring-opening dioxygenase ligb subunit | 325.63 | * | * |
| PADG_05827 | Arginine biosynthesis bifunctional protein argj | 476.16 | * | * |
| PADG_03149 | Aminopeptidase | 627.32 | * | * |
| PADG_08304 | Acetolactate synthase small subunit | 2250.36 | * | * |
| PADG_02719 | NAD dependent epimerase dehydratase family protein | 441.59 | 0.52 | 0 |
| PADG_08262 | Asparagine synthetase | 556.4 | 0.64 | 0 |
| PADG_03825 | NAD specific glutamate dehydrogenase | 1963.79 | * | * |
| PADG_01305 | Argininosuccinate lyase | 28.45 | * | * |
| PADG_00888 | Argininosuccinate synthase | 3230.81 | 0.66 | 0 |
| PADG_01404 | Aspartate aminotransferase | 1681.94 | * | * |
| PADG_01615 | Homocitrate synthase | 2480.23 | * | * |
| PADG_06289 | Lysine decarboxylase-like protein | 3085.26 | * | * |
| PADG_00875 | Pentafunctional AROM polypeptide | 534.7 | * | * |
| PADG_05058 | Chorismate mutase | 1627.34 | * | * |
| PADG_05896 | D 3-phosphoglycerate dehydrogenase | 740.06 | * | * |
| PADG_05301 | Cystathionine beta synthase | 683.38 | * | * |
| PADG_00349 | Kynureninase | 607.61 | * | * |
| PADG_06756 | Histidine biosynthesis trifunctional protein | 938.05 | * | * |
| PADG_08604 | ATP phosphoribosyltransferase | 1222.73 | * | * |
| PADG_03167 | 3-isopropylmalate dehydratase | 540.24 | * | * |
| **Nitrogen and sulfur metabolism** | |  |  |  |
| PADG_00446 | Oxidoreductase 2 nitropropane dioxygenase family | 3313.83 | * | * |
| PADG_08569 | Thiosulfate sulfurtransferase | 744.46 | 0.60 | 0 |
| **Nucleotide metabolism** | |  |  |  |
| PADG_00209 | Guanine deaminase | 321.64 | * | * |
| PADG_00322 | Xanthine phosphoribosyltransferase 1 | 4746.3 | * | * |
| PADG_01653 | Ribose phosphate pyrophosphokinase | 675.7 | * | * |
| PADG_05747 | Phosphoribosylformylglycinamidine synthase | 457.03 | * | * |
| PADG_06297 | Phosphoribosylamine glycine ligase | 617.21 | * | * |
| PADG_07780 | Phosphoribosylglycinamide formyltransferase | 601.88 | * | * |
| PADG_04099 | Bifunctional purine biosynthesis protein ADE17 | 9141.72 | 0.55 | 0 |
| PADG_07585 | Inosine 5 monophosphate dehydrogenase IMD2 | 2588.95 | 0.57 | 0 |
| PADG_01100 | Uracil phosphoribosyltransferase | 1640.11 | * | * |
| PADG_05321 | Mitochondrial nuclease | 1014.33 | * | * |
| PADG_00780 | Ribose phosphate pyrophosphokinase | 1879.31 | * | * |
| **C-compounds metabolism** | |  |  |  |
| PADG_03118 | Aldose 1-epimerase family protein | 862.54 | 0.53 | 0 |
| PADG_03943 | Phosphomannomutase | 1212.41 | * | * |
| PADG_08386 | Glycosyltransferase | 541.65 | * | * |
| PADG_03984 | Glucosamine fructose 6 phosphate aminotransferase | 1156.98 | * | * |
| PADG_07435 | Sorbitol utilization protein SOU2 | 2634.62 | 0.59 | 0 |
| PADG_02132 | Mannose 6 phosphate isomerase | 583.58 | * | * |
| PADG_04374 | UTP glucose 1 phosphate uridylyltransferase | 2352.22 | * | * |
| PADG_01486 | Short chain dehydrogenase reductase family | 623.71 | * | * |
| PADG_03278 | Myo inositol 1 phosphate synthase | 648.34 | * | * |
| **Signal transduction** | |  |  |  |
| PADG_01480 | Mitogen activated protein kinase MKC1 | 535.56 | * | * |
| PADG_03544 | Ser Thr protein phosphatase family protein | 618.15 | * | * |
| PADG_02212 | Serine threonine protein phosphatase PPT1 | 1134.79 | * | * |
| PADG_08399 | GTP binding protein ypt3 | 3662.66 | * | * |
| PADG_01243 | Rab GDP dissociation inhibitor | 6219.94 | * | * |
| PADG_04810 | GTP binding nuclear protein GSP1 Ran | 19149.31 | 0.30 | 0 |
| PADG_02800 | Adenylyl cyclase associated protein | 472.62 | * | * |
| **Transcription and transcriptional control** | |  |  |  |
| PADG_07689 | Transformer-SR ribonucleoprotein | 595.58 | * | * |
| PADG_06734 | U6 snrna binding protein | 927.95 | * | * |
| PADG_06768 | Rrna 2 O methyltransferase fibrillarin | 534.02 | * | * |
| PADG_05034 | RNA binding domain-containing protein | 1736.7 | * | * |
| PADG_06856 | Small nuclear ribonucleoprotein Sm D2 115 aa | 528.54 | * | * |
| PADG_03733 | Poly rc binding protein | 1011.45 | * | * |
| PADG_02473 | Pirin | 694.39 | * | * |
| PADG_07134 | Histone H4 | 2253.29 | * | * |
| PADG_07884 | Polyadenylate binding protein | 1003.83 | 0.58 | 0 |
| **Mitotic cell cycle and cell cycle control** | |  |  |  |
| PADG_01796 | Caffeine-induced death protein Cid2 | 552.99 | * | * |
| PADG_05893 | Nucleosome assembly protein | 2676.57 | * | * |
| PADG_05907 | Histone H2B type 1 A | 2731.64 | * | * |
| PADG_05931 | Condensin | 506.43 | * | * |
| **Glycolysis** | |  |  |  |
| PADG_01896 | Phosphoglycerate kinase | 10933.7 | 0.20 | 0 |
| PADG_03813 | Hexokinase | 1005.71 | * | * |
| PADG_00192 | 6 phosphofructokinase | 1158.94 | * | * |
| PADG_00668 | Fructose bisphosphate aldolase | 19990.67 | 0.37 | 0 |
| **Tricarboxylic acid** | |  |  |  |
| PADG_02063 | Pyruvate dehydrogenase E1 component subunit alpha | 1202.79 | * | * |
| PADG_02805 | Isocitrate dehydrogenase subunit 2 | 1872.1 | * | * |
| PADG_03977 | Isocitrate dehydrogenase subunit 1 | 3357.02 | 0.51 | 0 |
| PADG_00317 | Succinyl coa ligase subunit beta | 1074.65 | * | * |
| PADG_08387 | Citrate synthase | 7729.15 | 0.36 | 0 |
| **Glyoxalate cycle** | |  |  |  |
| PADG_04702 | Malate synthase | 2960.63 | * | * |
| **Methylcitrate cycle** | |  |  |  |
| PADG_04710 | 2 methylcitrate synthase | 17679.08 | 0.28 | 0 |
| **Electron transport and respiration** | |  |  |  |
| PADG_02578 | ATP synthase subunit 4 | 596.35 | * | * |
| PADG_07042 | ATP synthase subunit 5 | 1549.49 | * | * |
| PADG_07081 | Electron transfer flavoprotein subunit alpha | 1315.88 | 0.59 | 0 |
| PADG_02669 | Electron transfer flavoprotein subunit beta | 2895.44 | * | * |
| PADG_03872 | NADH cytochrome b5 reductase | 955.33 | 0.57 | 0 |
| PADG_08394 | Cytochrome b c1 complex subunit 2 | 720.82 | * | * |
| **Lipid, fatty acid and isoprenoid metabolism** | |  |  |  |
| PADG_00254 | Fatty acid synthase subunit alpha reductase | 1110.48 | * | * |
| PADG_00255 | Fatty acid synthase subunit beta dehydratase | 2244.05 | * | * |
| PADG_03449 | Isopentenyl diphosphate Delta isomerase | 807.48 | * | * |
| PADG_08651 | Peroxisomal multifunctional enzyme | 1142 | * | * |
| PADG_06876 | 3-hydroxyisobutyryl coa hydrolase | 3141.18 | * | * |
| PADG_01687 | 3-ketoacyl coa thiolase | 5137.5 | 0.47 | 0 |
| PADG_01677 | Acetyl coenzyme A synthetase | 655.5 | * | * |
| **Biosynthesis of cofactor and vitamins** | |  |  |  |
| PADG_00443 | Dihydropteroate synthase | 1062.11 | * | * |
| PADG_01897 | Chlorocatechol 1 2 dioxygenase | 857.03 | * | * |
| PADG_03944 | 26S proteasome non atpase regulatory subunit 10 | 27.87 | * | * |
| **Cell Growth and morphogenesis** | |  |  |  |
| PADG_05538 | Actin | 1639.2 | * | * |
| PADG_07249 | Actin binding protein | 1135.38 | 0.57 | 0 |
| PADG_05341 | Fimbrin | 604.95 | * | * |
| PADG_00128 | Tubulin alpha chain | 665.15 | * | * |
| PADG_05491 | GMF family protein, actin depolymerization factor | 2939.5 | 0.66 | 0.03 |
| PADG_03959 | ARP2 3 actin organizing complex subunit Sop2 | 1031.25 | 0.63 | 0 |
| PADG_07930 | ARP2 3 complex 20 kda subunit | 1695.34 | * | * |
| PADG_07733 | ARP2 3 complex 34 kda subunit | 661.72 | * | * |
| **Protein synthesis** | |  |  |  |
| PADG_04057 | Translation initiation factor eif3 | 4000.27 | * | * |
| PADG_00932 | Translation initiation factor eif3 | 478.38 | * | * |
| PADG_01891 | Translation initiation factor RLI1 | 656.19 | * | * |
| PADG_06110 | Translation factor SUI1 | 3845.67 | * | * |
| PADG_02896 | Elongation factor 1 beta | 27699.38 | 0.36 | 0 |
| PADG_06265 | Elongation factor 1 gamma 1 | 21636.54 | 0.41 | 0 |
| PADG_08125 | Elongation factor 2 | 8384.21 | 0.45 | 0 |
| PADG_00692 | Elongation factor 1 alpha | 25477.84 | 0.23 | 0 |
| PADG_02759 | Ribosome recycling factor domain-containing protein | 1902.46 | * | * |
| PADG_02752 | 116 kda U5 small nuclear ribonucleoprotein component | 110.37 | 0.53 | 0 |
| PADG_04730 | Nascent polypeptide associated complex subunit alpha | 1887.05 | * | * |
| PADG_03431 | Putative trna-binding protein | 533.64 | * | * |
| PADG_03440 | Prolyl trna synthetase | 454.75 | * | * |
| PADG_01558 | Histidyl tRNA synthetase | 1005.73 | * | * |
| PADG_02484 | Valyl tRNA synthetase | 844.82 | * | * |
| PADG_03689 | Tyrosyl tRNA synthetase | 1451.04 | * | * |
| PADG_05848 | Glycyl tRNA synthetase | 600.14 | * | * |
| PADG_05897 | Seryl tRNA synthetase | 2707.68 | * | * |
| PADG_08472 | Lysyl tRNA synthetase | 857.74 | * | * |
| PADG_04962 | Aspartyl tRNA synthetase | 2972.06 | 0.54 | 0 |
| PADG_00785 | Ribosomal protein S15 | 1042.95 | * | * |
| PADG_01503 | 37S ribosomal protein Rsm24 | 553.96 | * | * |
| PADG_04866 | 40S ribosomal protein S10 A | 3053.9 | * | * |
| PADG_02445 | 40S ribosomal protein S15 | 10623.49 | 0.39 | 0 |
| PADG_06502 | 40S ribosomal protein S20 | 7199.8 | 0.48 | 0 |
| PADG_06599 | 40S ribosomal protein S25 | 539.48 | * | * |
| PADG_08605 | 40S ribosomal protein S28 | 4991.9 | * | * |
| PADG_04848 | 60S ribosomal protein L8 B | 14038.4 | 0.63 | 0 |
| PADG_02828 | 60S ribosomal protein l10a | 1216.32 | 0.59 | 0 |
| PADG_07803 | 60S ribosomal protein L12 | 9790.37 | 0.63 | 0 |
| PADG_06726 | 60S ribosomal protein L17 | 2919.98 | 0.66 | 0 |
| PADG_01026 | 60S ribosomal protein L43 | 7369.92 | 0.66 | 0 |
| **Protein folding** | |  |  |  |
| PADG_00050 | T complex protein 1 subunit alpha | 1353.14 | * | * |
| PADG_00928 | T complex protein 1 subunit gamma | 1340.39 | * | * |
| PADG_02057 | T complex protein 1 subunit theta | 1461.5 | * | * |
| PADG_03441 | T complex protein 1 subunit alpha | 604.66 | * | * |
| PADG_08048 | T complex protein 1 subunit beta | 981.88 | * | * |
| PADG_08234 | T complex protein 1 subunit eta | 444.18 | * | * |
| PADG_08484 | T complex protein 1 subunit epsilon | 827.58 | * | * |
| **Protein targeting, sorting and translocation** | |  |  |  |
| PADG_04034 | Mitochondrial protein import protein MAS5 | 441.38 | * | * |
| PADG_00187 | Snare ykt6 | 739.12 | * | * |
| PADG_00326 | ADP ribosylation factor | 638.49 | * | * |
| **Protein modification and assembly** | |  |  |  |
| PADG_00602 | HNRNP arginine N methyltransferase | 874.79 | * | * |
| **Protein degradation** | |  |  |  |
| PADG_06851 | 26S proteasome non atpase regulatory subunit 11 | 693.35 | * | * |
| PADG_00599 | 26S protease regulatory subunit 6A | 946.17 | * | * |
| PADG_00631 | Proteasome 26S subunit | 2324.02 | * | * |
| PADG_03192 | Proteasome subunit alpha type 5 A | 1071.37 | * | * |
| PADG_04067 | Proteasome component PUP3 | 3288.96 | 0.61 | 0 |
| PADG_03424 | Ubiquitin-activating enzyme E1 | 737.43 | 0.66 | 0 |
| PADG_04167 | Aspartyl aminopeptidase | 888.45 | * | * |
| PADG_08265 | Methionine aminopeptidase | 680.33 | * | * |
| **Transport** | |  |  |  |
| PADG_06033 | NIPSNAP family protein | 3382.2 | 0.33 | 0 |
| PADG_08176 | Phosphatidylinositol phosphatidylcholine transfer protein | 1546.75 | * | * |
| PADG_06378 | Carnitine acyl carnitine carrier | 486.8 | * | * |
| PADG_08368 | Cofilin | 4118.88 | 0.33 | 0 |
| PADG_00622 | Arsenical pump driving atpase | 1226.15 | * | * |
| PADG_01847 | Stomatin family protein | 582.14 | * | * |
| PADG_01440 | ADP ATP carrier protein | 1281.41 | * | * |
| **Cell Rescue, Defense and Virulence** | |  |  |  |
| PADG_07674 | Carbonic anhydrase | 1294.52 | 0.64 | 0 |
| PADG_00324 | Peroxisomal catalase | 7532.22 | 0.54 | 0 |
| PADG_04587 | Peroxiredoxin HYR1 | 424.23 | * | * |
| PADG_04912 | Ahpc/TSA-thioredoxin peroxidase/alkyl hydroperoxide reductase | 5662 | 0.61 | 0 |
| PADG_07946 | Peroxisomal matrix protein | 6803.02 | 0.63 | 0 |
| PADG_02030 | Hsp90 co chaperone Cdc37 | 1582.78 | * | * |
| PADG_00765 | Heat shock protein HSP98 | 987.2 | * | * |
| PADG_03927 | DNAJ domain containing protein | 691.37 | * | * |
| PADG_08118 | Hsp70 like protein | 62668.96 | 0.66 | 0 |
| PADG_07715 | Heat shock protein | 10535.23 | 0.50 | 0 |
| PADG_02761 | Heat shock protein SSB1 | 1560.43 | * | * |
| PADG_01711 | Hsp90 co chaperone AHA1 | 2746.85 | 0.54 | 0 |
| **Unclassified** | |  |  |  |
| PADG_06709 | Zinc binding oxireductase domain containing protein | 424.27 | * | * |
| PADG_00541 | Hypothetical protein | 1679.04 | * | * |
| PADG_03031 | Cobw domain containing protein | 1372.54 | * | * |
| PADG_03121 | DUF757 domain containing protein | 583.55 | * | * |
| PADG_03203 | BAR domain protein | 751.09 | * | * |
| PADG_03244 | HD domain containing protein | 1493.64 | * | * |
| PADG_07355 | Conserved hypothetical protein | 27.87 | * | * |
| PADG_07412 | DUF1479 domain-containing protein | 897.69 | * | * |
| PADG_07670 | SAP domain protein | 689.63 | * | * |
| PADG_08480 | Conserved hypothetical protein | 585.72 | * | * |
| PADG_05701 | Conserved hypothetical protein | 338.45 | 0.02 | 0 |

^1^ Accession number obtained in the *Paracoccidioides* database available at <http://www.broadinstitute.org/annotation/genome/paracoccidioides_brasiliensis/MultiHome.html>.

^2^ PLGS score is the result of different mathematical models for peptide and fragment assign prediction. Acceptable score values consider protein identification with a minimum confidence level of 95% and a false discovery rate of 4%.

^3^ Fold change values were obtained by dividing the values of protein abundance (in fmol) from *P. brasiliensis* yeast cells during macrophage infection by the abundance in control**.** Proteins with a minimum fold change of 50% were considered regulated.

^4^ *p*-value of the fold change. It were considered statistically significant the p-values less than 0.05 and higher than 0.95.

* Proteins detected in *P. brasiliensis* *Pb*18 only in the control condition.
