# Supplementary material for: Macrophage Interaction with Paracoccidioides brasiliensis Yeast Cells Modulates Fungal Metabolism and Generates a Response to Oxidative Stress
Source: PLoS One. 2015 Sep 11;10(9):e0137619. doi: 10.1371/journal.pone.0137619 (PMC4567264; doi:10.1371/journal.pone.0137619)
Supplement: S3 File — PepFrag1 and PepFrag2 correspond to the peptides matches compared to the database by PLGS, VarMod corresponds to variable modifications, In Source corresponds to fragmentation that occurred in the ionization source, Missed Cleavage indicates the missed cleavage performed by trypsin and Neutral loss HO and NH correspond to water and ammonia precursor losses. (PDF) [file pone.0137619.s003.pdf]

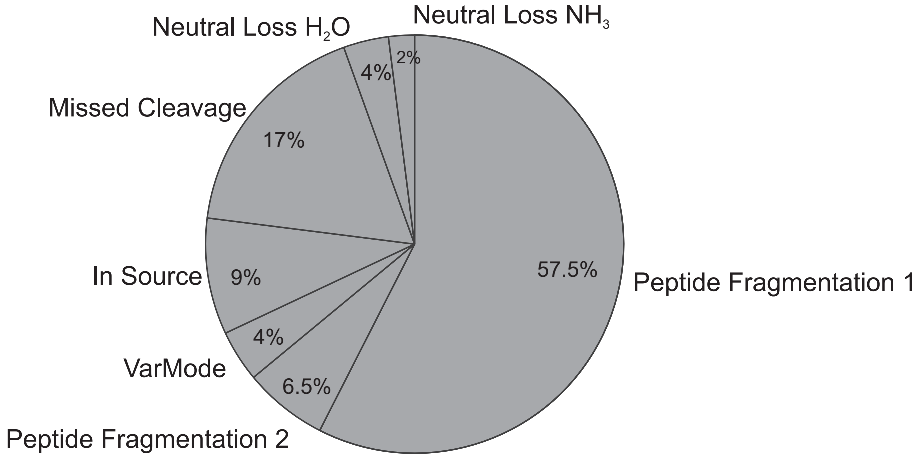

Supplementary Figure 1. NanoUPLC-MSE data quality analysis. PepFrag1 and PepFrag2 correspond to the peptides matches compared to the database by PLGS, VarMod corresponds to variable modifications, In Source corresponds to fragmentation that occurred in the ionization source, Missed Cleavage indicates the missed cleavage performed by trypsin and Neutral loss HO and NH correspond to water and ammonia precursor losses.
