# Supplementary material for: Macrophage Interaction with Paracoccidioides brasiliensis Yeast Cells Modulates Fungal Metabolism and Generates a Response to Oxidative Stress
Source: PLoS One. 2015 Sep 11;10(9):e0137619. doi: 10.1371/journal.pone.0137619 (PMC4567264; doi:10.1371/journal.pone.0137619)
Supplement: S4 File — The number of identified fragments according to the error range (x-axis). (PDF) [file pone.0137619.s004.pdf]

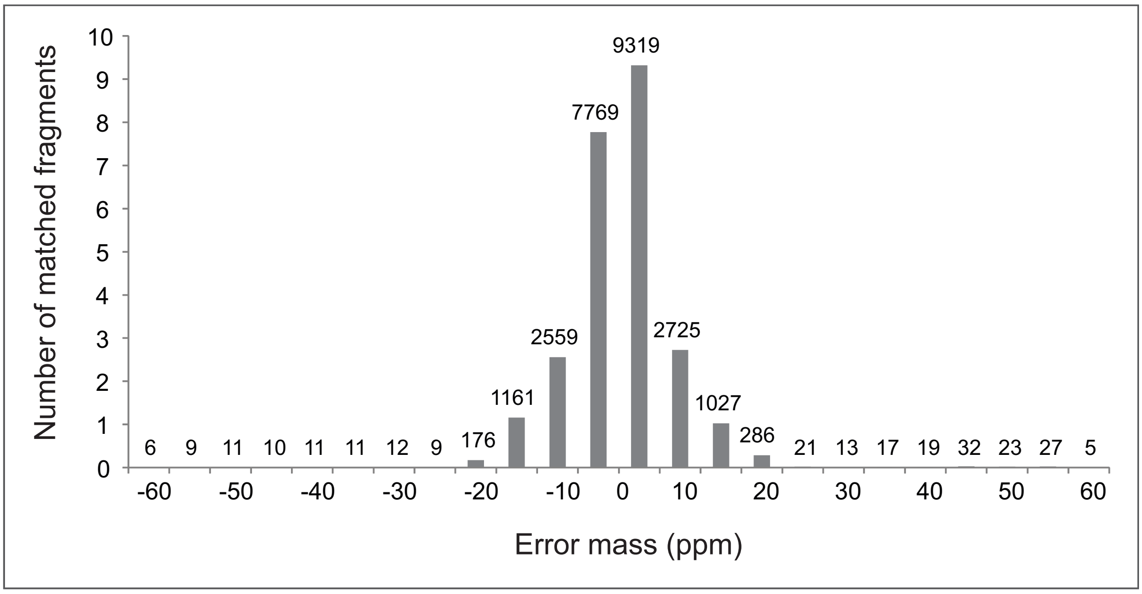


**Supplementary Figure 2. Mass error of the identified fragments.** The number of identified fragments according to the error range (x-axis).
