# Supplementary material for: Macrophage Interaction with Paracoccidioides brasiliensis Yeast Cells Modulates Fungal Metabolism and Generates a Response to Oxidative Stress
Source: PLoS One. 2015 Sep 11;10(9):e0137619. doi: 10.1371/journal.pone.0137619 (PMC4567264; doi:10.1371/journal.pone.0137619)
Supplement: S5 File — Quantified fragments were sorted according to the fragment amount (Fmol) and plotted in the graphics as grey circles. Standard protein was indicated by red circle. A protein with a low coefficient of variance between samples was used to normalize the expression data and allow comparisons of the control and P. brasiliensis data from infected macrophage. (PDF) [file pone.0137619.s005.pdf]

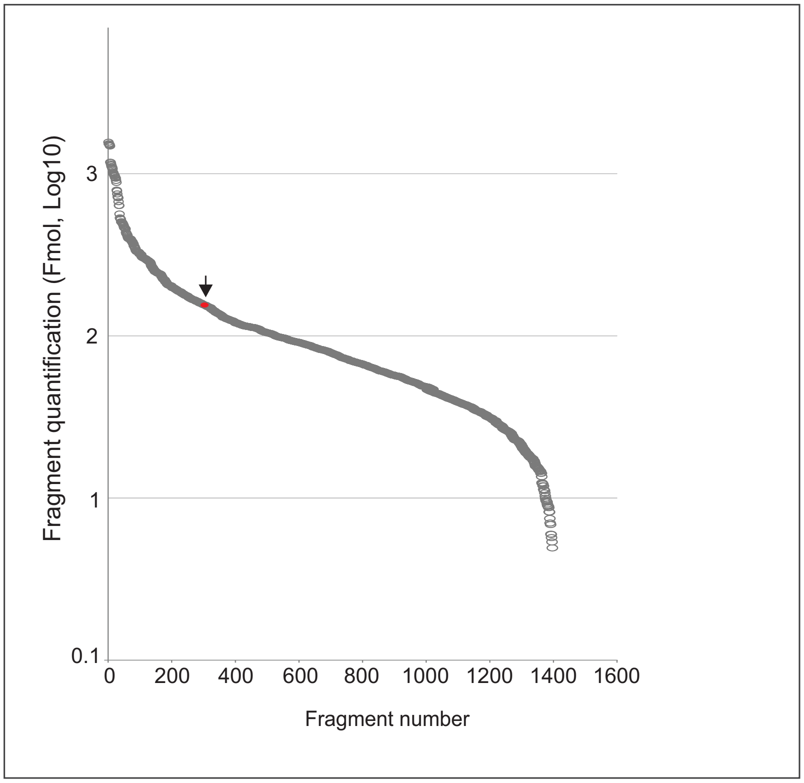

Supplementary Figure 3. Detection dynamic range. Quantified fragments were sorted according to the fragment amount (Fmol) and plotted in the graphics as grey circles. Standard protein was indicated by red circle. A protein with a low coefficient of variance between samples was used to normalize the expression data and allow comparisons of the control and *P. brasiliensis* data from infected macrophage.
