# Supplementary material for: Macrophage Interaction with Paracoccidioides brasiliensis Yeast Cells Modulates Fungal Metabolism and Generates a Response to Oxidative Stress
Source: PLoS One. 2015 Sep 11;10(9):e0137619. doi: 10.1371/journal.pone.0137619 (PMC4567264; doi:10.1371/journal.pone.0137619)
Supplement: S6 File — (A) Abundance (%) of upregulated P. brasiliensis proteins during the interaction with macrophages in agreement with their biological functions. (B) Abundance (%) of down-regulated P. brasiliensis proteins during the interaction with macrophages in agreement with their biological functions. (PDF) [file pone.0137619.s006.pdf]

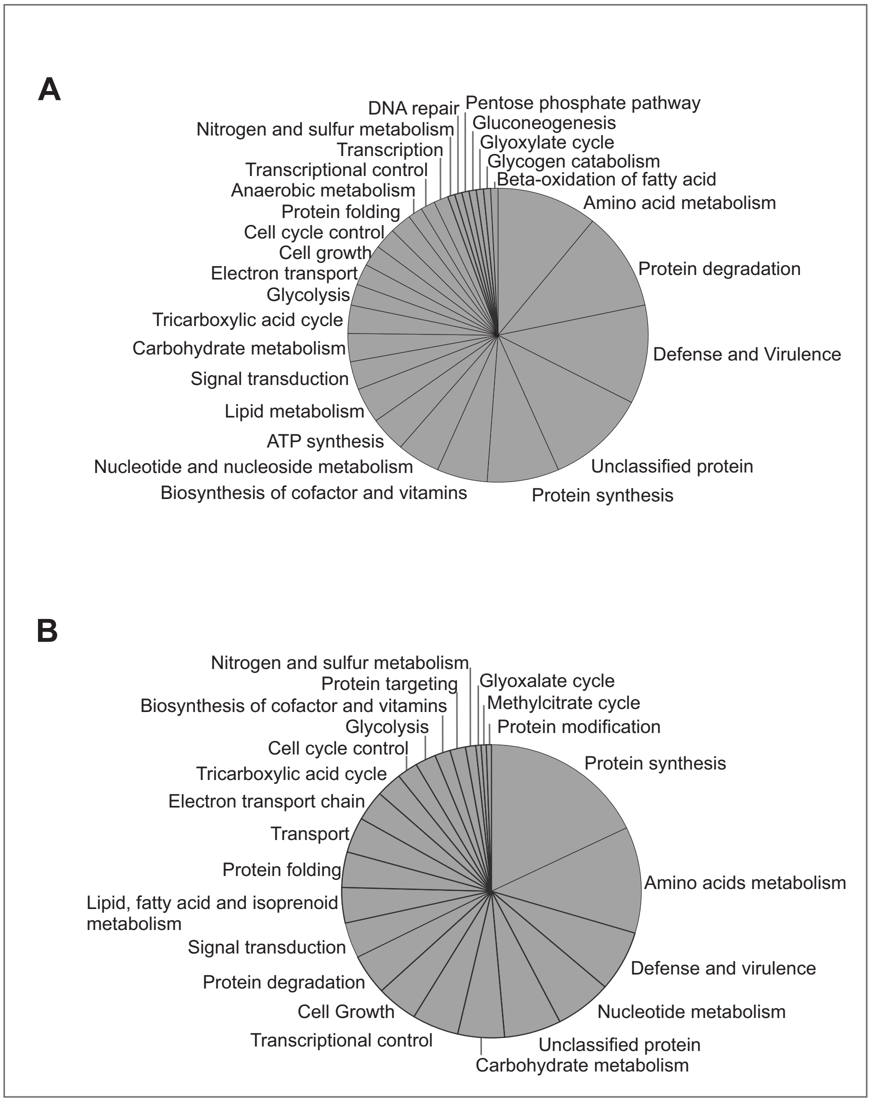

Supplementary Figure 4: Functional categorization of *P. brasiliensis*-regulated proteins during macrophage infection. (A) Abundance (%) of upregulated *P. brasiliensis* proteins during the interaction with macrophages in agreement with their biological functions. (B) Abundance (%) of down-regulated *P. brasiliensis* proteins during the interaction with macrophages in agreement with their biological functions.
