# Supplementary material for: Macrophage Interaction with Paracoccidioides brasiliensis Yeast Cells Modulates Fungal Metabolism and Generates a Response to Oxidative Stress
Source: PLoS One. 2015 Sep 11;10(9):e0137619. doi: 10.1371/journal.pone.0137619 (PMC4567264; doi:10.1371/journal.pone.0137619)
Supplement: S7 File — Transcript levels of genes encoding fructose 1,6 biphosphatase (pbase) and cytochrome c peroxidase (ccp). Transcript levels were measured using quantitative RT-PCR. Data were normalized to the beta tubulin protein transcript and are presented as fold change calculated based on the rate of macrophage interaction to control condition. The Student's t-test was used for statistical comparisons. Error bars represent the standard deviation from three biological replicates, while * represents p≤0.05. (PDF) [file pone.0137619.s007.pdf]

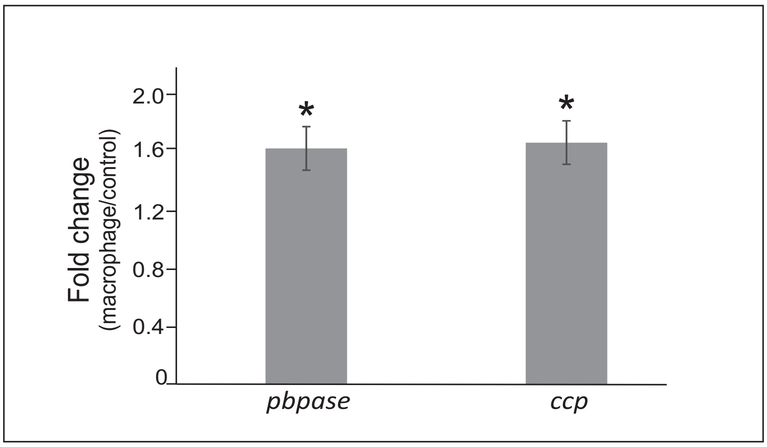

Supplementary Figure 5. Quantification of transcripts encoding proteins that were up-regulated during macrophage infection. Transcript levels of genes encoding fructose 1,6 biphosphatase (pbase) and cytochrome c peroxidase (ccp). Transcript levels were measured using quantitative RT-PCR. Data were normalized to the beta tubulin protein transcript and are presented as fold change calculated based on the rate of macrophage interaction condition to control condition. The Student's t-test was used for statistical comparisons. Error bars represent the standard deviation from three biological replicates, while * represents $p \leq 0.05$.
